# Programmed cell death-driven remodeling of the melanoma microenvironment enables prognostic stratification and therapeutic prediction

**DOI:** 10.3389/fimmu.2025.1612217

**Published:** 2025-08-20

**Authors:** Bo Hu, Shengnan Chai, Xuan Li, Qiang Zhang, Mei Jin, Long Zhang

**Affiliations:** Wound Healing Center, Peking University Third Hospital, Beijing, China

**Keywords:** melanoma, programmed cell death, prognostic model, immunotherapy, tumor microenvironment

## Abstract

**Background and objective:**

Melanoma exhibits profound biological complexity, driven by immune evasion, phenotypic plasticity, and resistance to therapy. While programmed cell death (PCD) shapes tumor–immune interactions, its mechanistic landscape in melanoma remains incompletely defined. This study aims to comprehensively characterize PCD-related signatures and their associations with tumor heterogeneity, prognosis, and immunotherapeutic outcomes.

**Methods:**

Single-cell RNA sequencing data from melanoma cohorts (cutaneous and acral subtypes) were used to assess PCD activity via AUCell-based scoring across major cell types. Cell-type–specific analyses examined heterogeneity, metabolic dependencies, and pathway correlations. Intercellular communication was analyzed using CellChat. Bulk RNA sequencing data were then integrated to identify PCD-related gene signatures, and machine learning models (LASSO, Ridge, XGBoost) were applied to develop a prognostic model. Immune infiltration, immunogenomic correlations, and immunotherapy responses were further evaluated using ESTIMATE, CIBERSORT, TMB, IPS, and external ICB-treated cohorts.

**Results:**

Among all cell types, melanoma cells exhibited the highest PCD activation, with disulfidptosis, immunogenic cell death (ICD), and autosis being the most prominent. High PCD activity was linked to advanced clinical stage, lymphatic metastasis, and poor prognosis. Melanoma subpopulations with hyperactivated PCD displayed elevated copy number variation (CNV) burden, enhanced fibroblast/endothelial interactions, and invasive transcriptional profiles. A 15-gene prognostic signature was developed, effectively stratifying survival and immunotherapy response across multiple cohorts. Low-risk tumors demonstrated favorable immune infiltration (CD8^+^ T cells, M1 macrophages), higher tumor mutational burden (TMB), and greater immunogenicity, while high-risk tumors exhibited immune exclusion, cancer-associated fibroblast (CAF) enrichment, and adverse mutations.

**Conclusion:**

This study highlights the functional and clinical significance of PCD heterogeneity in melanoma and provides a validated prognostic model for patient stratification and therapeutic decision-making. These findings underscore the potential of targeting PCD dynamics as a novel approach in melanoma management.

## 1 Introduction

Melanoma, arising from malignant transformation of epidermal melanocytes, remains the most fatal cutaneous malignancy due to its high metastatic proclivity, immune evasion, and resistance to conventional therapies (1, 2). Despite substantial progress in early diagnosis and the clinical deployment of immune checkpoint inhibitors (ICIs) targeting CTLA-4, PD-1, and PD-L1, melanoma continues to exhibit pronounced inter- and intra-tumoral heterogeneity, therapeutic refractoriness, and frequent dissemination at distant sites, contributing to dismal outcomes in advanced-stage disease (3, 4). Cutaneous melanoma (CM) and acral melanoma (AM) are clinically and molecularly distinct subtypes. CM typically arises on sun-exposed skin and harbors ultraviolet (UV)-induced mutational signatures (5), while AM originates on glabrous sites (palms, soles, nail beds), lacks UV-associated mutations, and is more prevalent in non-Caucasian populations (6). Irrespective of subtype, melanoma displays aggressive clinical behavior and an immunologically evasive phenotype that complicates durable treatment response (7). These features underscore the urgent need to dissect the molecular and cellular mechanisms underpinning melanoma progression, therapeutic resistance, and immune escape.

Programmed cell death (PCD) encompasses a diverse array of genetically regulated cellular demise mechanisms—including apoptosis, necroptosis, ferroptosis, pyroptosis, immunogenic cell death (ICD), and more recently characterized modalities such as autosis and disulfidptosis (8, 9). These distinct PCD modalities are increasingly recognized not only as markers of cellular stress and damage but also as integral components of tumor biology, capable of shaping the tumor immune microenvironment (TME) through modulation of antigen release, cytokine production, and immune cell crosstalk (10, 11). In the context of melanoma, where immune evasion and immunoediting are tightly interwoven with tumor progression, the interplay between PCD and TME remodeling assumes particular relevance (12). Yet, the functional significance, transcriptional regulation, and cell-type–specific engagement of these death pathways remain incompletely understood. Whether PCD activity delineates vulnerability, immune interaction, or aggressive tumor phenotypes across heterogeneous melanoma contexts remains an open question, particularly at single-cell resolution.

Given the complexity of melanoma biology and the expanding landscape of therapeutically actionable cell death programs, a comprehensive characterization of PCD heterogeneity is urgently warranted. Such investigation may illuminate previously unrecognized associations between specific death modalities and tumor aggressiveness, immune dynamics, or therapeutic resistance. Furthermore, elucidating the interplay between PCD activation and melanoma progression holds promise for identifying prognostic biomarkers and informing the development of cell death–modulating strategies tailored to distinct biological subtypes.

## 2 Materials and methods

### 2.1 Single-cell transcriptomic processing and quantification of cell death modalities

The melanoma single-cell RNA sequencing dataset (GSE215120) was processed using the Seurat R package (v4.3.0). After log-normalization and feature selection, dimensionality reduction was performed using principal component analysis (PCA) and Uniform Manifold Approximation and Projection (UMAP). Graph-based clustering was conducted via the shared nearest neighbor (SNN) algorithm at a resolution of 0.5. The marker genes used for cell type annotation were referenced from the original publication of the dataset (13). To evaluate the transcriptional activity of regulated cell death pathways at single-cell resolution, 21 curated gene sets representing distinct cell death modalities were assessed using the AUCell algorithm (v1.18.1). Differential activation of cell death pathways across treatment conditions, anatomical compartments, and clinical variables was assessed using non-parametric Wilcoxon or Kruskal–Wallis tests implemented via ggpubr (v0.6.0), with multiple-testing correction applied. Associations between cell death activity and overall survival (OS, in months) were quantified using Spearman correlation, and results were visualized with ComplexHeatmap (v2.16.0) and circlize (v0.4.15). All graphical representations were generated using ggplot2 (v3.4.4), with statistical annotations performed using ggsignif (v0.6.4).

### 2.2 Subtype-specific assessment of cell death pathways in stromal and immune compartments

To investigate the functional relevance of PCD programs in melanoma-associated stromal and immune microenvironments, we performed subtype-level analyses in fibroblasts, T cells, and NK cells—three major cell populations that exhibit extensive interaction with malignant cells, as inferred from prior intercellular communication profiling. Subclustering within each compartment was performed using the Seurat workflow, including NormalizeData(), FindVariableFeatures(), ScaleData(), and RunPCA(). To correct for batch effects, we applied Harmony integration across patient samples. Downstream dimension reduction and clustering were performed using RunUMAP(), FindNeighbors(), and FindClusters(), with the resolution parameter set to 0.3 to balance subtype granularity with interpretability. To further refine subtype assignment and enhance functional interpretability, we implemented module scoring using the AddModuleScore() function (14, 15). Subtype-specific gene signatures were compiled for each cell type, and per-cell module scores were computed to quantify the expression enrichment of these gene sets. Average scores across clusters were then used to assist in resolving ambiguous cluster identities and to support subtype annotations. This scoring-based refinement was particularly useful in distinguishing transcriptionally similar subpopulations and ensured robust subtype identification without altering the underlying clustering structure. For cell subtype annotation, we applied marker gene sets derived from canonical sources, recent high-resolution studies (16–27) internal expert curation, and the CellMarker database (http://bio-bigdata.hrbmu.edu.cn/CellMarker).

AUCell scores were recalculated within each subset using AUCell for the 21 PCD modalities previously curated. For downstream analyses, cell death programs exhibiting either a significant positive correlation with OS (i.e., NETosis and oxeiptosis) or consistently low activation across all cell types (i.e., excitotoxicity) were excluded. Subtype-level pathway activity comparisons were performed using ggpubr (v0.6.0), with Wilcoxon rank-sum tests used for two-group comparisons and Kruskal–Wallis tests applied when comparing more than two subtypes.

To elucidate the biological correlates of cell death activity, we performed Spearman correlation analyses between AUCell-derived activity scores and functional pathway enrichment scores across malignant cells. Pathway scores were calculated using single-sample Gene Set Variation Analysis (ssGSEA) implemented via the GSVA R package (v1.48.3). Gene sets from the Molecular Signatures Database (MSigDB) were used as input gene sets. Correlation matrices were visualized using the ComplexHeatmap and ggplot2 packages.

### 2.3 Stratification of melanoma cells based on cell death activity and downstream molecular characterization

To delineate the relationship between cell death activity and transcriptional heterogeneity within melanoma cells, we first applied AUCell to calculate enrichment scores for 18 PCD modalities across individual melanoma cells derived from the GSE215120 single-cell dataset. Thirteen PCD modalities were retained based on their prognostic relevance and population-wide activation patterns. To interrogate global PCD activity within melanoma cells, we performed single-cell level quantification of enrichment scores across 13 discrete PCD pathways using the AUCell algorithm. For each pathway, individual cells were stratified into high- and low- activity states according to whether their AUCell-derived enrichment scores exceeded or fell below the median value, a commonly employed thresholding approach in high-dimensional single-cell transcriptomic analyses to delineate biologically distinct cellular phenotypes. A chi-squared test was performed to evaluate the statistical significance of differences in melanoma cell abundance between high and low groups.

To obtain robust PCD-based transcriptional states, we further intersected the high-activity and low-activity cells across all 13 modalities. Cells were assigned to the Melanoma_High group if they exhibited above-median AUCell-derived enrichment scores across all 13 curated PCD modalities, indicating uniformly elevated pathway activity. Conversely, cells with below-median scores across all 13 PCD programs were categorized as Melanoma_Low, thereby representing a globally quiescent PCD phenotype. Subsequent analyses—including differential gene expression (FindMarkers()), Gene Ontology enrichment (via clusterProfiler, v4.10.0), pathway scoring (via GSVA, v1.48.3), and copy number inference (via inferCNV, v1.14.0)—were conducted to characterize molecular, functional, and genomic distinctions between the two populations. Clinical metadata—including primary tumor site, tumor stage, and patient age—were integrated to assess phenotypic divergence between Melanoma_High and Melanoma_Low subpopulations, utilizing the ggpubr, ggalluvial, and ComplexHeatmap packages for visualization and stratified comparison.

### 2.4 Inference of ligand–receptor-mediated intercellular communication patterns

To systematically infer intercellular signaling networks between transcriptionally defined melanoma subpopulations, we employed the CellChat R package (v1.6.1). The normalized gene expression matrix from melanoma cells and all non-malignant cells was used as input, and cells were annotated based on their original cell type classification. Ligand–receptor interactions were predicted using the curated CellChatDB.human database, and communication probabilities were computed under the “truncated mean” method with default thresholds.

Separate CellChat objects were constructed for the Melanoma_High and Melanoma_Low subsets to enable comparative analyses of outgoing and incoming signaling strength, global communication probability matrices, and pathway-specific contributions. Differential signaling roles (sender, receiver, mediator, influencer) were quantified using the netAnalysis_signalingRole_heatmap and netAnalysis_contribution functions.

Pathway-specific communication strength was visualized across VEGF, CSPG4, COLLAGEN, CXCL, TGFβ, PDGF, MIF, GALECTI and NOTCH axes. Dot plots and circle plots were generated using netVisual_aggregate and netVisual_bubble. In addition, ligand–receptor pair analysis was conducted to pinpoint subtype-specific enrichment using the subsetCommunication and netVisual_individual functions, with statistical filtering (p < 0.05) applied to highlight biologically relevant interactions.

### 2.5 Construction and evaluation of a cell death–linked prognostic model

Differential gene expression analysis between the Melanoma_High and Melanoma_Low subgroups was conducted using the limma package (v3.52.4) in R. To identify melanoma-specific genes, we integrated normal skin transcriptomic data from the Genotype-Tissue Expression (GTEx) project, a large-scale resource providing RNA-seq data across multiple normal human tissues, with melanoma samples from TCGA-SKCM. Batch effects between GTEx and TCGA data were corrected using the sva package (v3.44.0) via the ComBat algorithm. Overlapping differentially expressed genes (DEGs) were identified using the VennDiagram (v1.7.3) and dplyr (v1.1.2) packages.

Univariate Cox regression analysis was conducted with the survival package (v3.5-5) to assess associations between gene expression and OS. A total of 101 machine learning model combinations were systematically constructed by pairing multiple feature selection algorithms (e.g., LASSO, CoxBoost, BART, GBM, PLSRcox) with diverse modeling algorithms (e.g., Ridge regression, Random Forest, Elastic Net, Supervised Principal Components), using packages including glmnet (v4.1-8), CoxBoost (v1.5), randomForestSRC (v3.1.1), plsRcox (v1.5.5), gbm (v2.1.8), superpc (v1.09), BART (v2.9), and survivalsvm (v0.1-2). Each model was trained in the TCGA-SKCM cohort and externally validated using GSE65904. The optimal model was selected based on the highest average concordance index (C-index), calculated via survcomp (v1.46.0).

Feature reduction and regularization were performed using LASSO Cox regression via glmnet. L1-penalized Cox proportional hazards modeling was used to optimize gene selection, with the λ penalty parameter tuned through 10-fold cross-validation. Patients were assigned individual risk scores calculated as a weighted linear combination of gene expression levels and corresponding LASSO-derived coefficients. Survival stratification was assessed using Kaplan–Meier curves and log-rank tests. Time-dependent ROC curves were generated using timeROC (v0.4) to evaluate predictive performance at 1-, 3-, and 5-year intervals. Discrimination ability was further quantified by C-index using survcomp.

A multivariate nomogram integrating risk scores and clinicopathologic variables was constructed using rms (v6.2) to visualize individualized OS probabilities. Calibration curves were used to assess model prediction accuracy. Gene expression heatmaps and risk distribution plots were constructed using pheatmap, and visual styling was finalized with ggplot2 and ComplexHeatmap. External validation of the model in GSE65904 was conducted identically to ensure methodological reproducibility.

To evaluate the independent prognostic value of the risk score in melanoma, both univariate and multivariate Cox proportional hazards regression analyses were conducted using the survival and survminer R packages. Clinical variables, including tumor site, stage, AJCC_T, AJCC_N, AJCC_M, race, gender, age, and tissue type, were incorporated into the analysis. Hazard ratios (HRs) with 95% confidence intervals (CIs) were calculated to quantify effect sizes, and p-values < 0.05 were considered statistically significant. Variables that were significant in univariate analysis (p < 0.05) were then included in the multivariate model to assess the independent prognostic significance of the risk score, along with other clinical factors. This approach was applied to both the TCGA-SKCM and GSE65904 cohorts.

### 2.6 Immune cell infiltration analysis and tumor microenvironment profiling

Immune cell infiltration was assessed using six deconvolution algorithms: MCPcounter, CIBERSORT, QUANTISEQ, EPIC, CIBERSORT-ABS, and TIMER. These tools estimated immune cell proportions from melanoma gene expression data. Visualizations were generated with ggplot2, including heatmaps and bar plots for immune infiltration patterns. Survival analysis was conducted using the survival and survminer packages, with Kaplan–Meier curves to assess the correlation between immune infiltration, as derived from CIBERSORT results, and OS. The Estimation of Stromal and Immune cells in Malignant Tumors using Expression data (ESTIMATE) algorithm was employed to calculate tumor purity, immune, and stromal scores. Gene expression of immune checkpoint and immunomodulatory genes was analyzed, with clusterProfiler used for Gene Set Enrichment Analysis (GSEA) to identify enriched pathways in the low- and high-risk groups.

### 2.7 Mutation-related analysis and ICI response modeling

To explore the immune landscape and therapeutic potential of the cell death–associated risk model, we analyzed distinct tumor microenvironment features in the TCGA-SKCM cohort. Cancer-associated fibroblast (CAF) abundance was estimated using the EPIC algorithm as implemented in the immunedeconv R package (v2.0.4). Immune exclusion was evaluated independently using two distinct metrics: (1) the Exclusion score, derived from the original TIDE framework and extracted from published TCGA-SKCM results, and (2) the Merck18 signature, an 18-gene expression-based surrogate for immune resistance to anti–PD-1 therapy. Merck18 composite scores were computed using the GSVA package (v1.44.5). Microsatellite instability (MSI) status was retrieved from UCSC Xena clinical annotation data. Three external datasets were analyzed to assess the predictive validity of the risk model in immune checkpoint blockade (ICB) contexts: IMvigor210 (anti–PD-L1–treated urothelial carcinoma), GSE35640 (anti–CTLA-4), and GSE78220 (anti–PD-1). Individual risk scores were computed using coefficients from the 15-gene prognostic signature via the glmnet package. Tumor mutational burden (TMB) was computed as the total number of non-synonymous mutations per megabase from TCGA MAF files. Somatic mutation analyses and visualizations were performed using the maftools package (v2.14.0), and survival comparisons of frequently mutated genes were evaluated using the log-rank test. Immunophenoscore (IPS) values were obtained from The Cancer Immunome Atlas (TCIA) and stratified by CTLA4/PD1 combinatorial status. IPS distributions between risk groups were compared using ggpubr and ggplot2.

### 2.8 Drug sensitivity prediction and melanoma diagnosis

Drug sensitivity was predicted using the oncoPredict R package (v0.2), leveraging GDSC2 database expression and drug response data for model training. Pre-processed melanoma expression data underwent batch correction, and low-variance genes (variance < 0.2) were excluded. Drug response predictions were visualized with heatmaps and violin plots. To assess the link between the 15-gene signature and melanoma progression, we retrieved AJCC_T, N, M, and overall stage data from TCGA-SKCM. Patients were stratified by model-derived risk scores, and clinical stage distributions were compared across risk groups to evaluate the model’s capacity to reflect tumor burden. Afterwards, Spearman correlation assessed the relationship between 15 prognostic genes and 13 PCD modalities in GSE215120, with AUCell used to quantify cell death activities, visualized via corrplot and ComplexHeatmap. GO enrichment analysis of the 15-gene panel was performed using the clusterProfiler package. Gene symbols were converted to Entrez IDs via the org.Hs.eg.db database. Significantly enriched terms were defined as those with Benjamini–Hochberg adjusted *p*-values (p.adjust) < 0.05. Cell expression patterns were visualized via UMAP using the Seurat package.

An XGBoost classifier, trained on GSE215120 data, distinguished melanoma cells based on 15 signature genes. Model performance was evaluated using AUC, optimized with 5-fold cross-validation and interpreted with SHAP values via the SHAPforxgboost package. To confirm generalizability, the trained XGBoost classifier was applied to GSE222446. Data preprocessing, UMAP visualization, and prediction probability scoring were performed identically. Classification performance was again assessed via AUC. Top-ranked diagnostic features in the external dataset were evaluated using the same SHAP interpretation framework.

### 2.9 Statistical analysis

All statistical analyses were performed using R. Survival associations were evaluated via univariate and multivariate Cox regression using the survival and survminer packages, with Kaplan–Meier curves and log-rank tests for visualization and comparison. Immune infiltration and microenvironmental features were quantified using six deconvolution algorithms, with between-group differences assessed by Wilcoxon rank-sum tests. TMB, IPS, and ESTIMATE scores were similarly compared. Drug sensitivity predictions were conducted using oncoPredict, and SHAP values from SHAPforxgboost identified top gene contributors. AUCell-derived cell death activity scores were correlated with GSVA-based pathway enrichment using Spearman’s method. To address multiple hypothesis testing, differential expression analyses were adjusted using the Benjamini–Hochberg method to control the false discovery rate (FDR), with significance thresholds set at FDR < 0.05 and |log_2_FC| > 0.5. Pathway enrichment analyses performed with clusterProfiler also incorporated FDR correction by default. In comparisons involving multiple cell subtypes or gene sets (e.g., violin plots), pairwise tests were conducted using Wilcoxon tests with FDR correction applied when multiple comparisons were made in parallel.

## 3 Results

### 3.1 Cell death pathway activation landscape in melanoma microenvironment and clinical correlations

**Figure 1:**
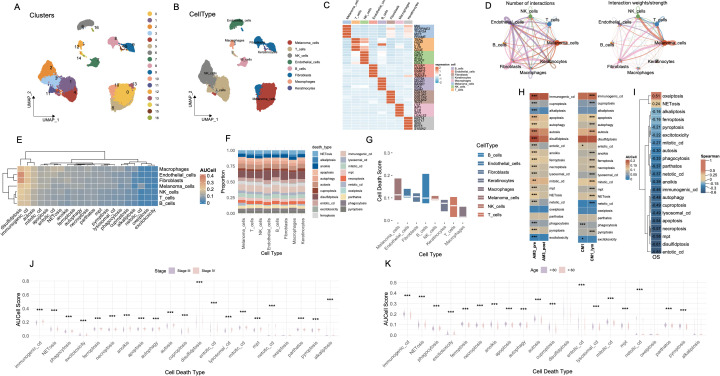
Cell-type interactions and cell death profiles across different conditions. **(A)** UMAP visualization of 16 distinct cell clusters identified from single-cell RNA-seq data. Each cluster is color-coded, representing transcriptionally distinct cell populations. **(B)** The distribution of cell types, including melanomas, T cells, NK cells, B cells, fibroblasts, macrophages, and keratinocytes, in the data. **(C)** The expression of key marker genes across different cell types, highlighting genes characteristic of each population. **(D)** The number and strength of interactions between cell types, with stronger interactions indicated by thicker and more intense lines. **(E)** Heatmap of AUCell scores for various cell death pathways across different cell types. **(F)** The proportion of each cell type involved in different death mechanisms. **(G)** Bar plot showing cell death scores for each cell type across different pathways. **(H)** Comparison of cell death activity between AM3_pre and AM3_post, and CM1 and CM1_lym groups. **(I)** Spearman’s correlation matrix showing the relationship between cell death pathways and OS. **(J)** Boxplot comparing the AUCell scores for different cell death types, stratified by disease stage (Stage III vs. Stage IV). **(K)** Boxplot analysis of AUCell scores for various cell death types across age groups. ***P<0.001. UMAP, Uniform Manifold Approximation and Projection; AUCell, Area Under the Curve cell; OS, Overall Survival.

The workflow of this study is illustrated in **Supplementary Figure S1**. Utilizing the melanoma single-cell RNA sequencing dataset (GSE215120), we delineated distinct cellular clusters and performed precise annotations of major cell lineages employing UMAP dimensionality reduction methodologies (**Figures 1A, B**, **Supplementary Figures S2A-F**). Subsequent marker gene expression profiling robustly corroborated the identities of diverse cell populations, encompassing melanoma cells, macrophages, T cells, NK cells, endothelial cells, fibroblasts, keratinocytes, and B cells (**Figure 1C**). Comprehensive intercellular communication analyses revealed extensive and complex crosstalk between melanoma cells and key components of the tumor microenvironment, particularly immune and stromal compartments such as NK cells, T cells, and fibroblasts (**Figure 1D**).

We systematically evaluated the functional activation landscape of diverse cell death modalities across all identified cellular subsets utilizing AUCell scoring metrics (**Figure 1E**). The gene sets corresponding to the 21 types of PCD are presented in **Supplementary Table S1**. Among all cell types, disulfidptosis exhibited the highest activation levels, followed closely by immunogenic cell death (ICD), whereas oxeiptosis demonstrated the lowest degree of functional activation. Proportional analyses of cell types further substantiated these differential activations across death modalities (**Figure 1F**). Collectively, melanoma cells, endothelial cells and fibroblasts demonstrated elevated median scores across cell death pathways (**Figure 1G**).

AM3_post samples (post-anti-PD1 therapy) and CM1_lym samples (lymphatic metastatic lesions) were separately extracted for subsequent comparative analyses (**Supplementary Figures S2J-L**). Following anti-PD1 therapeutic intervention (AM3_post), salient alterations in cell death pathway activities emerged relative to pre-treatment baseline conditions (AM3_pre). Except for oxeiptosis and pyroptosis, all other cell death modalities exhibited significantly decreased activation levels (p < 0.001). Furthermore, comparative evaluations between lymphatic metastatic lesions (CM1_lym) and primary neoplasms (CM1) unveiled significantly elevated activities across the majority of cell death modalities in lymphatic metastatic tissues, with the exceptions of phagocytosis, excitotoxicity, and entotic cell death, which exhibited higher activation in primary tumor tissues (**Figure 1H**).

Spearman correlation analyses delineated significant associations between specific cell death pathways and patient OS, notably implicating oxeiptosis and NETosis in positive correlations with prolonged OS durations, while the remaining cell death modalities exhibited inverse relationships (**Figure 1I**). Stratified assessments based on clinical staging disclosed pronounced variations in cell death pathway engagement, with all cell death modalities displaying significantly heightened activities in Stage IV malignancies compared to Stage III (**Figure 1J**). Moreover, most cell death pathways were significantly more active in patients younger than 60 years of age (p < 0.001) (**Figure 1K**).

### 3.2 Cell death pathway activation in fibroblast, T cell, and NK cell subtypes

We conducted an exhaustive analysis of the functional activation landscapes of various cell death modalities across the three cell types most intimately interacting with melanoma cells, including fibroblasts, T cells, and NK cells. It merits particular attention that cell death modalities that were positively correlated with OS—specifically NETosis and oxeiptosis—as well as those exhibiting consistently low activation levels across cell populations, such as excitotoxicity, were excluded from subsequent analyses. The marker genes used for cell subtype annotation were manually curated based on well-established lineage-specific signatures reported in high-impact immunology and oncology studies. The complete gene list is provided in **Supplementary Table S2**.

The fibroblast compartment comprises five molecularly and functionally distinct subtypes. Pan-cancer-associated fibroblasts (PanCAFs), marked by ACTA2, COL1A1, COL1A2, COL3A1, CIR, CIS, PDPN, PDGFRB, and SERPINF1 expression (19–21, 23, 25), represent a conserved population driving tumor progression through ECM remodeling, stromal signaling, and immune evasion across diverse malignancies. Matrix CAFs (mCAFs), characterized by APOD, CCL11, COL1A1, COL1A2, COL3A1, POSTN, MMP14, FNI, LUM, and CTHRC1 expression (19, 24), establish the dense fibrotic stroma typical of desmoplastic tumors through specialized extracellular matrix deposition and tissue stiffening. Transitional features between mesothelial and mesenchymal lineages define mesothelial CAFs (mesCAFs), with ANXA1, ANXA2, BDKRB1, CALB2, CCDC80, CFB, CRABP2, CXCL1, CXCL6, and S100A10 expression (19) contributing to peritoneal dissemination and stem-like niche formation. Antigen-presenting CAFs (apCAFs) uniquely express MHC class II molecules (CD74, HLA-DRA, HLA-DRB1) alongside chemokines CCL21 and CXCL12 while lacking classical co-stimulatory molecules (20, 22), potentially inducing T cell tolerance through partial antigen presentation capability. Localized to perivascular niches, vascular CAFs (vCAFs) regulate angiogenesis via ADIRF, RGS5, SPARCL1, CRIP1, NDUFA4L2, MCAM, MYH11, PDK4, FABP4, and TINAGL1 expression (20), supporting endothelial function and vessel maturation through specialized fibroblast-endothelial crosstalk.

**Figure 2:**
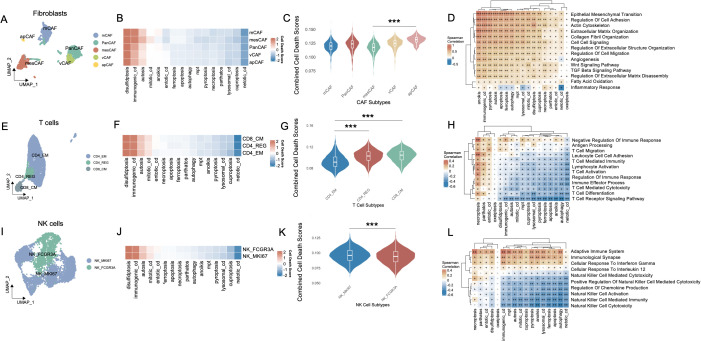
Cell-type interactions, cell death activity, and pathway enrichment across different immune cell subtypes. **(A)** UMAP visualization of fibroblast subtypes, including PanCAFs, mcCAFs, mesCAFs, apCAFs, and vCAFs, identified based on single-cell RNA-seq data. Each fibroblast subtype is color-coded to represent transcriptionally distinct populations. **(B)** Heatmap of cell death pathway scores across different fibroblast subtypes. **(C)** Violin plot comparing the combined cell death scores across fibroblast subtypes. **(D)** Spearman’s correlation matrix showing the relationship between cell death pathways and various fibroblast-enriched pathways. **(E)** UMAP visualization of T cell subtypes, including CD4_EM, CD4_REG, and CD8_CM, with distinct clusters of each T cell population identified in the data. **(F)** Heatmap of cell death pathway scores across CD4_EM, CD4_REG, and CD8_CM subtypes. **(G)** Violin plot comparing the combined cell death scores among T cell subtypes. **(H)** Pathway enrichment analysis for T cell subtypes, focusing on T cell-specific pathways and their relationship with PCD. **(I)** UMAP visualization of NK cell subtypes, including NK_MKI67 and NK_FCGR3A, with distinct clusters of these NK populations identified in the data. **(J)** Heatmap showing the cell death pathway scores across NK cell subtypes. **(K)** Violin plot comparing combined cell death scores between NK_MKI67 and NK_FCGR3A subtypes. **(L)** Pathway enrichment analysis for NK cell subtypes, focusing on NK cell-specific pathways and their relationship with PCD. Positive correlations are shown in brown and negative correlations in blue, and “+” symbols denote correlation strength based on coefficient (ρ) thresholds: “+”: weak correlation (0 < ρ ≤ 0.3); “++”: moderate correlation (0.31 ≤ ρ ≤ 0.6); “+++”: strong correlation (ρ > 0.6). ***P<0.001. UMAP, Uniform Manifold Approximation and Projection; CAF, Cancer-Associated Fibroblast; PanCAF, Pan-cancer Cancer-Associated Fibroblast; mCAF, Matrix Cancer-Associated Fibroblast; mesCAF, Mesothelial Cancer-Associated Fibroblast; apCAF, Antigen-presenting Cancer-Associated Fibroblast; vCAF, Vascular Cancer-Associated Fibroblast; CD4_EM, CD4^+^ Effector Memory T cell; CD4_REG, CD4^+^ Regulatory T cells; CD8_CM, CD8^+^ Central Memory T cell; NK, Natural Killer; NK_MKI67, MKI67^+^ Proliferative Natural Killer cell; NK_FCGR3A, FCGR3A^+^ Cytotoxic Natural Killer cell; PCD, Programmed Cell Death.

Among the various cell death modalities, disulfidptosis exhibited the most pronounced activation across fibroblast subtypes, whereas netotic cell death demonstrated comparatively subdued activity (**Figure 2B**). Notably, apCAFs exhibited significantly higher activation scores, whereas mesCAFs demonstrated the lowest levels of activation, as illustrated in **Figure 2C** (p < 0.001). These cell death modalities showed robust and statistically significant positive correlations with several pathways intrinsically linked to CAFs, including epithelial-mesenchymal transition, regulation of cell adhesion, extracellular matrix organization, collagen fibril organization, and cell-cell signaling (**Figure 2D**). In T cells, three transcriptionally distinct subpopulations were identified: CD4^+^ effector memory T cells (CD4_EM), CD4^+^ regulatory T cells (CD4_REG), and CD8^+^ central memory T cells (CD8_CM) (27) (**Figure 2E**). The heatmap (**Figure 2F**) illustrates the heterogeneous activation patterns of PCD pathways across these subtypes. Violin plot analysis (**Figure 2G**) revealed that CD8_CM T cells exhibited the highest combined PCD activity, followed by CD4_REG and CD4_EM cells. Notably, both CD8_CM and CD4_REG subsets showed significantly elevated death scores compared to CD4_EM cells (p < 0.001). Subsequent investigations revealed that the activation of these cell death modalities in these subsets was significantly negatively correlated with the majority of T cell-associated signaling pathways, including T cell receptor signaling, T cell activation, T cell differentiation, and immune response regulation (**Figure 2H**). Similarly, in NK cells, distinct subpopulations—NK_FCGR3A and NK_MKI67—exhibited markedly divergent activation landscapes across multiple cell death pathways (**Figures 2I, J**). NK_FCGR3A represents a cytotoxic and terminally differentiated NK subset involved in antibody-dependent cellular cytotoxicity, whereas NK_MKI67 reflects a proliferative and cycling phenotype, suggestive of active expansion in response to microenvironmental cues (26). NK_MKI67 cells demonstrated substantially elevated activation of a broad spectrum of cell death programs (p < 0.001) (**Figure 2K**). Moreover, the activation of cell death modalities was found to be significantly and inversely associated with key functional signatures of NK cell-mediated immunity (**Figure 2L**).

### 3.3 Melanoma cells stratified by cell death activity exhibit distinct molecular and clinical characteristics

**Figure 3:**
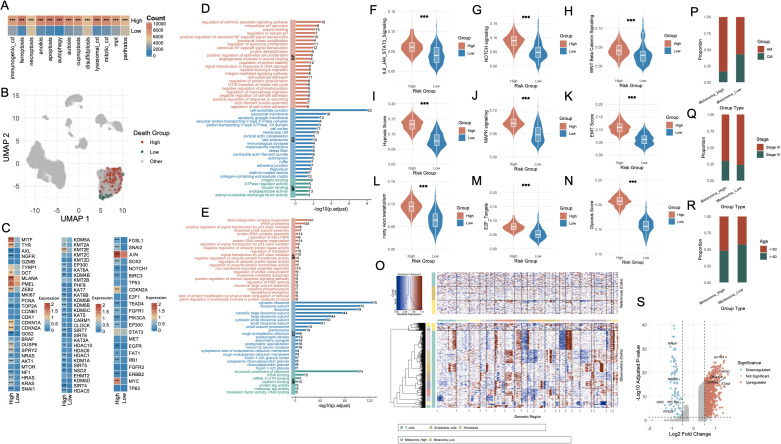
Characterization of melanoma subgroups based on PCD-related features and molecular phenotypes. **(A)** Bar plot showing the number of melanoma cells with high versus low AUCell scores for each PCD type. **(B)** UMAP visualization highlighting the single-cell distribution of melanoma cells stratified by death score groups (High, Low, Other). **(C)** Heatmap displaying differential expression of representative genes between Melanoma_High and Melanoma_Low groups, including lineage-defining genes, cell cycle regulators, and transcription factors. **(D, E)** Functional enrichment analysis of upregulated genes in Melanoma_High (red) and Melanoma_Low (blue) groups. **(F–N)** Violin plots comparing key signaling and phenotypic scores between Melanoma_High and Melanoma_Low groups. **(O)** Chromatin accessibility profiles from single-cell ATAC-seq showing distinct open chromatin landscapes in Melanoma_High vs. Melanoma_Low cells, with clustering by cell type. **(P–R)** Bar plots illustrating associations between Melanoma_High and Melanoma_Low groups with clinical annotations: melanoma subtype (AM vs. CM), tumor stage (Stage II vs. III), and patient age (<60 vs. ≥60). **(S)** Volcano plot highlighting differentially expressed genes between Melanoma_High and Melanoma_Low groups, with notable upregulated and downregulated genes labeled. *P<0.05; **P<0.01; ***P<0.001. PCD, Programmed Cell Death; AUCell, Area Under the Curve cell; UMAP, Uniform Manifold Approximation and Projection; ATAC-seq, Assay for Transposase-Accessible Chromatin using sequencing; AM, Acral Melanoma; CM, Cutaneous Melanoma.

To interrogate the relationship between cell death activity and melanoma cell states, we stratified melanoma cells based on the AUCell scores of each cell death modality, dichotomized by median values into high- and low-activity groups. Across 13 distinct cell death types (**Supplementary Figures S3A-M**), the number of melanoma cells within the high-activity group was consistently and significantly greater than that in the low-activity group (P < 0.001, **Figure 3A**). Subsequently, we extracted melanoma cells that simultaneously belonged to either the high group or low group across all 13 cell death modalities. This yielded two transcriptionally distinct populations: Melanoma_High (n = 895) and Melanoma_Low (n = 89), visualized via UMAP projection in **Figure 3B**.

Differential expression analysis revealed striking transcriptomic differences between the two groups (**Figure 3C**). The first panel highlights melanoma-associated genes, including MITF, AXL, NGFR, TYRP1, and DCT, which are involved in the regulation of melanoma cell identity, dedifferentiation, and invasive potential. The second panel presents genes associated with histone modification, such as KDM5A, KMT2A, EZH2, and members of the HDAC family, which participate in chromatin remodeling and epigenetic control of gene expression. The third panel displays genes related to transcriptional and post-transcriptional regulation, including TP53, NOTCH1, and SOX2, reflecting distinct regulatory landscapes between the Melanoma_High and Melanoma_Low populations. GO enrichment analysis of Melanoma_High revealed significant upregulation of biological programs associated with extrinsic apoptotic signaling, NF-κB signaling, inflammatory response and TNF signaling (**Figure 3D**, **Supplementary Table S3**). In contrast, Melanoma_Low cells were enriched for pathways including RNA splicing, mRNA catabolic process, protein targeting to ER, ribosome biogenesis, and translational initiation, indicating enhanced engagement in basal biosynthetic and translational machinery (**Figure 3E**, **Supplementary Table S4**).

To further characterize these transcriptional states, we assessed the activity of key oncogenic and tumor progression pathways. Violin plot analyses showed that IL6-JAK-STAT3, NOTCH, WNT, MAPK, EMT, E2F targets, and oxidative stress signatures were significantly upregulated in the Melanoma_High group (p < 0.001, **Figures 3F–N**). CNV profiling further substantiated these findings (**Figure 3O**). Melanoma_High cells exhibited markedly greater genomic instability, with widespread chromosomal gains and losses across multiple regions, particularly in loci associated with chromatin organization and oncogenic amplification, whereas Melanoma_Low cells maintained a relatively copy-neutral profile. Clinical annotation of the two groups revealed distinct compositional biases. Melanoma_High cells were predominantly derived from AM samples (anti-PD1–treated), whereas Melanoma_Low cells were enriched in CM samples (**Figure 3P**). Additionally, Melanoma_High cells were more frequently associated with advanced clinical stage (Stage IV) (**Figure 3Q**) and younger patients (< 60 years) (**Figure 3R**). Differential expression analysis between the two groups yielded a large set of significantly dysregulated genes (**Figure 3S**).

### 3.4 Melanoma_high and melanoma_low cells exhibit divergent intercellular communication landscapes

**Figure 4:**
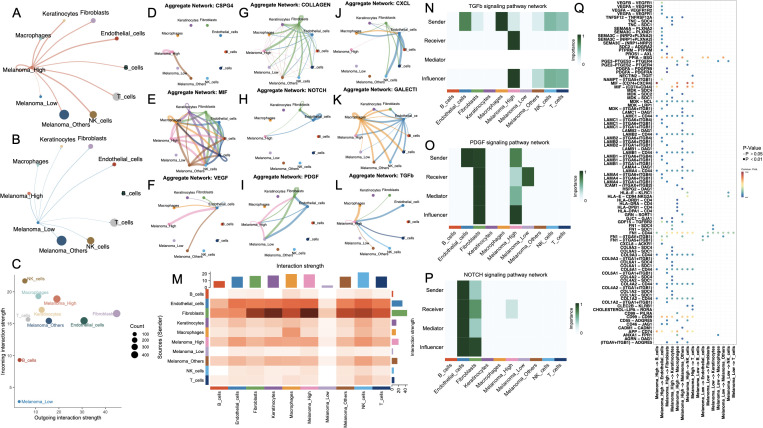
Cell-cell communication networks and key signaling pathways among melanoma subgroups and stromal/immune cell populations. **(A, B)** Circle plots showing the outgoing interaction networks from melanoma subgroups—**(A)** Melanoma_High and **(B)** Melanoma_Low—to surrounding stromal and immune cell types. Edge thickness represents the strength of inferred intercellular communication. **(C)** Scatter plot summarizing incoming versus outgoing interaction strength for each cell type; point size reflects total number of interactions. **(D–L)** Aggregate communication networks for selected ligand–receptor families: CSPG4, MIF, VEGF, COLLAGEN, NOTCH, PDGF, CXCL, GALECT1, and TGFB. **(M)** Heatmap summarizing overall interaction strengths between sender and receiver cell types. **(N–P)** Functional role analysis for TGFB, PDGF, and NOTCH signaling pathways, showing sender, receiver, mediator, and influencer roles across melanoma and stromal/immune cells. **(Q)** Dot plot showing top-ranked ligand–receptor pairs by importance score across cell-type pairs, colored by adjusted p-value.

To dissect the intercellular communication architecture underlying divergent cell death states, we systematically mapped ligand–receptor-mediated signaling interactions across melanoma subpopulations. Comparative analysis of global communication networks revealed that Melanoma_High cells engaged in substantially more extensive crosstalk than their Melanoma_Low counterparts, which remained largely inert within the signaling landscape (**Figures 4A, B**). Within the comprehensive interaction topology (**Figure 4C**), Melanoma_High cells exhibited elevated incoming signaling alongside moderate outgoing communication. In contrast, Melanoma_Low cells demonstrated uniformly diminished bidirectional signaling activity.

Pathway-specific communication maps (**Figures 4D–L**) revealed that Melanoma_High cells exhibited broad and intensified participation across key signaling networks, including VEGF, CSPG4, COLLAGEN, CXCL, and TGFB. Additionally, NOTCH, TGFβ, and PDGF signaling pathways were also significantly active in Melanoma_High, particularly with fibroblast and endothelial cells interactions. In contrast, Melanoma_Low cells showed sparse or absent involvement across nearly all networks, reflecting a signaling-inactive phenotype. Moreover, a comparison of system-wide intercellular signaling landscapes across all cell types demonstrated that Melanoma_High cells act as prominent signal senders and receivers, particularly engaging with fibroblasts and endothelial cells. In contrast, Melanoma_Low cells remained largely passive, with minimal engagement in outgoing or incoming communication.

To further delineate intercellular signaling dynamics, we prioritized three pivotal pathways—TGF-β, PDGF, and NOTCH—given their central roles in tumor progression and microenvironmental remodeling. All major cell types were functionally categorized as senders, receivers, mediators, or influencers within each pathway (**Figures 4N–P**). Notably, in the TGF-β signaling axis, Melanoma_High cells acted as both dominant senders and influencers, while Melanoma_Low cells exhibited minimal functional involvement, suggesting unidirectional paracrine regulation from aggressive melanoma states to less active subpopulations. In the PDGF network, Melanoma_High cells exhibited multifaceted involvement across all functional axes—acting as signal initiators, receivers, mediators, and influencers—whereas Melanoma_Low cells remained largely passive with modest receptor engagement. For the NOTCH pathway, signaling was predominantly orchestrated by fibroblasts and endothelial cells, with Melanoma_High cells weakly participating as downstream receivers, and Melanoma_Low cells exhibiting negligible involvement. Ligand–receptor pair analysis (**Figure 4Q**) revealed that Melanoma_High cells prominently engaged in pro-angiogenic and immunosuppressive signaling via enriched interactions such as VEGFA–VEGFR1, TGFB1–TGFBR1, MDK–LRP1, and JAG1–NOTCH1, which were largely absent in Melanoma_Low cells.

### 3.5 Development and validation of PCD–associated prognostic signature in melanoma

**Figure 5:**
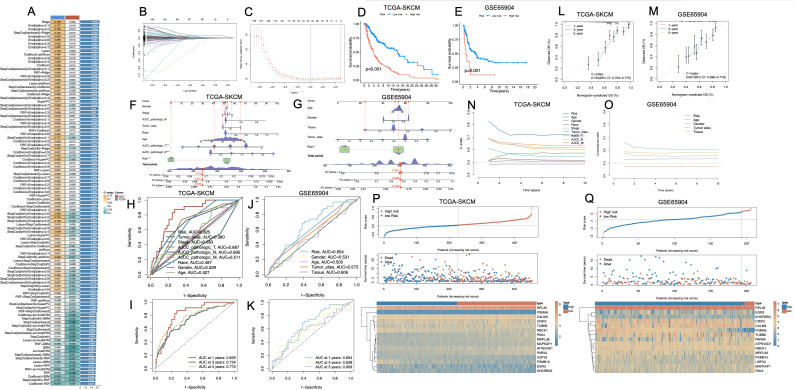
Construction and validation of a prognostic risk model based on melanoma cell–associated PCD-related genes. **(A)** Bar plot comparing multiple machine learning algorithms for prognostic model development across different cohorts (TCGA-SKCM, GSE65904), with C-index values indicating predictive performance. **(B, C)** LASSO regression analysis showing coefficient profiles **(B)** and optimal λ value selection by cross-validation **(C)**. **(D, E)** Kaplan–Meier survival curves showing significant differences in overall survival between high- and low-risk groups in the TCGA-SKCM **(D)** and GSE65904 **(E)** cohorts (p < 0.001). **(F, G)** Nomogram integrating risk score with clinical variables to predict survival in TCGA-SKCM **(F)** and GSE65904 **(G)**. **(H–K)** ROC curves for risk score and clinical parameters in TCGA-SKCM **(H, I)** and GSE65904 **(J, K)**, indicating predictive performance at 1, 3, and 5 years. **(L, M)** Calibration plots demonstrating consistency between predicted and observed survival in both cohorts. **(N, O)** Time-dependent concordance index curves for clinical features and the risk score, showing that the model maintains predictive value over time. **(P, Q)** Distribution of risk scores and survival status of patients, and heatmaps of PCD-related gene expression patterns between high- and low-risk groups in TCGA-SKCM **(P)** and GSE65904 **(Q)**. PCD, Programmed Cell Death; TCGA, The Cancer Genome Atlas; SKCM, Skin Cutaneous Melanoma; C-index, Concordance index; LASSO, Least Absolute Shrinkage and Selection Operator; ROC, Receiver Operating Characteristic.

To identify prognostically relevant gene features associated with PCD phenotypes in melanoma, we first performed differential gene expression analysis between the Melanoma_High and Melanoma_Low subgroups. By applying thresholds of |log_2_ fold change| > 0.5 and p < 0.05, we identified a total of 4,366 differentially expressed genes (DEGs). To further refine genes with disease specificity, we integrated normal skin tissue transcriptomes from the GTEx database with TCGA-SKCM samples, followed by batch effect correction and normalization. A second round of differential expression analysis between normal and melanoma samples yielded a set of DEGs, of which 981 genes overlapped with the 4,366 DEGs identified in the Melanoma_High vs. Low comparison. These intersecting genes were then subjected to univariate Cox regression analysis using OS data from TCGA-SKCM, resulting in 265 genes significantly associated with patient prognosis (p < 0.05; **Supplementary Table S5**). We benchmarked 101 combinations of machine learning models, integrating multiple algorithms and parameter settings, across TCGA-SKCM and GSE65904 datasets. Among all models, the Ridge regression algorithm achieved the highest concordance index (C-index) and was selected as the core modeling strategy (**Figure 5A**). Finally, we employed LASSO regression to optimize feature selection and minimize redundancy, ultimately generating a parsimonious 15-gene prognostic signature (**Figures 5B, C**). The risk score was calculated by integrating these coefficients into a weighted sum of gene expression values: Risk Score = (RPL35 × 0.000682144) + (PSMA6 × -0.088701462) + (TUBB6 × 0.002186538) + (CRIP2 × -0.000252985) + (ATP6V0D1 × 0.006421136) + (MRPL36 × 0.026186757) + (EGR3 × -0.006950893) + (RBCK1 × -0.011232856) + (MAPKAP1 × 0.019832427) + (USP33 × -0.013763014) + (PAK4 × 0.000140103) + (KHDRBS3 × -0.017767292) + (PSMB10 × -0.015051055) + (CALM3 × 0.004868883) + (PARVA × 0.012902798). Kaplan–Meier survival analyses revealed that patients stratified into the high-risk group exhibited significantly poorer OS in both TCGA-SKCM (p < 0.001, **Figure 5D**) and GSE65904 (p < 0.001, **Figure 5E**). Nomogram construction incorporating clinical parameters alongside the risk score (**Figures 5F, G**) enabled individualized survival prediction. Time-dependent ROC analysis demonstrated the model’s consistent prognostic performance over time, with AUCs of 0.825, 0.734, and 0.772 at 1, 3, and 5 years respectively in the TCGA cohort, and corresponding values of 0.654, 0.628, and 0.606 in the GSE65904 cohort (**Figures 5H-K**). Calibration curves confirmed the concordance between predicted and observed outcomes (**Figures 5L, M**). Notably, the C-index of the integrated nomogram surpassed conventional clinical variables alone (**Figures 5N, O**). Risk stratification plots (**Figures 5P, Q**) highlighted a distinct separation between high- and low-risk groups in terms of mortality distribution and expression heatmaps.

In the univariate Cox regression analysis of the TCGA-SKCM cohort, tumor site (HR = 0.358, 95% CI: 0.211–0.606, p < 0.001), clinical stage (HR = 1.430, 95% CI: 1.204–1.697, p < 0.001), AJCC_T (HR = 1.366, 95% CI: 1.203–1.551, p < 0.001), and AJCC_N (HR = 1.405, 95% CI: 1.212–1.629, p < 0.001) were all significantly correlated with OS. Race (HR = 0.452, 95% CI: 0.295–0.691, p < 0.001) and age (HR = 1.020, 95% CI: 1.009–1.031, p < 0.001) also exhibited prognostic relevance. Notably, the PCD–associated risk score emerged as the most powerful prognostic indicator, demonstrating a remarkably high hazard ratio (HR = 136.186, 95% CI: 50.250–369.091, p < 0.001). Upon multivariate adjustment, only AJCC_T (HR = 1.303, 95% CI: 1.138–1.491, p < 0.001), AJCC_N (HR = 1.596, 95% CI: 1.294–1.969, p < 0.001), and the risk score (HR = 114.699, 95% CI: 40.382–325.783, p < 0.001) remained independently associated with survival outcomes (**Table 1**).

**Table 1:** Univariate and multivariate Cox regression analysis of clinical variables and risk score in the TCGA-SKCM cohort.

Variable	Univariate analysis		Multivariate analysis	
	HR (95% CI)	P-value	HR (95% CI)	P-value
Tumor_site	0.358 (0.211-0.606)	<0.001	0.921 (0.522-1.628)	0.779
Stage	1.430 (1.204-1.697)	<0.001	0.953 (0.749-1.212)	0.696
AJCC_T	1.366 (1.203-1.551)	<0.001	1.303 (1.138-1.491)	<0.001
AJCC_N	1.405 (1.212-1.629)	<0.001	1.596 (1.294-1.969)	<0.001
AJCC_M	2.012 (0.887-4.561)	0.094	–	–
Race	0.452 (0.295-0.691)	<0.001	0.653 (0.416-1.023)	0.063
Gender	1.000 (0.725-1.379)	0.999	–	–
Age	1.020 (1.009-1.031)	<0.001	1.014 (1.003-1.025)	0.053
riskScore	136.186 (50.250-369.091)	<0.001	114.699 (40.382-325.783)	<0.001

In the GSE65904 cohort, univariate Cox regression analysis identified tumor site, tissue type, and risk score as significantly associated with OS (all p < 0.05). Upon multivariate adjustment, only the PCD–associated risk score remained an independent prognostic factor (HR = 10.781, 95% CI: 2.604–44.642, p < 0.01) (**Table 2**).

**Table 2:** Univariate and multivariate Cox regression analysis of clinical variables and risk score in the GSE65904 cohort.

Variable	Univariate analysis		Multivariate analysis	
	HR (95% CI)	P-value	HR (95% CI)	P-value
Tumor_site	1.425 (1.069-1.898)	<0.05	1.274 (0.947-1.716)	0.110
Tissue	1.604 (1.199-2.144)	<0.01	1.450 (1.037-2.027)	0.057
Gender	1.460 (0.932-2.288)	0.098	–	–
Age	0.999 (0.984-1.014)	0.881	–	–
riskScore	8.689 (2.280-33.111)	<0.01	10.781 (2.604-44.642)	<0.01

### 3.6 Immune landscape and tumor microenvironment characterization between high- and low-risk groups

**Figure 6:**
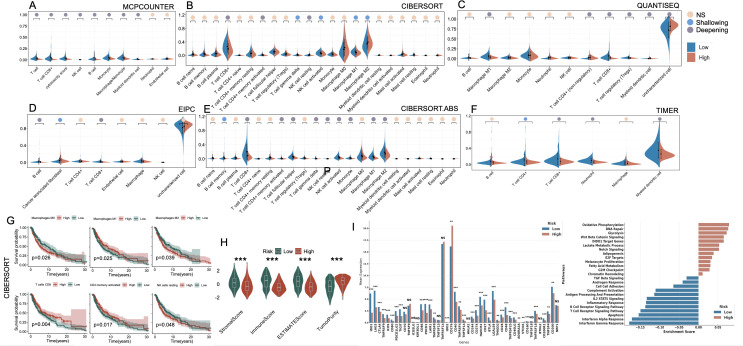
Immune landscape and functional characterization between high- and low-risk groups defined by PCD-associated gene signature. **(A–F)** Violin plots illustrating differences in immune cell infiltration between high-risk and low-risk groups across six computational deconvolution methods: **(A)** MCPcounter, **(B)** CIBERSORT, **(C)** QUANTISEQ, **(D)** EPIC, **(E)** CIBERSORT.ABS, and **(F)** TIMER. Significance is marked as NS (not significant), Shallowing (0.01 ≤ p < 0.05), or Deepening (p < 0.01) based on effect magnitude. **(G)** Kaplan–Meier survival analyses of key immune cell subsets derived from CIBERSORT, showing their impact on prognosis within high-risk and low-risk groups. **(H)** Comparison of StromalScore, ImmuneScore, ESTIMATEScore, and TumorPurity between high- and low-risk groups. **(I)** Expression levels of immune checkpoint and regulatory genes in high- vs. low-risk groups. **(J)** GSEA-based pathway enrichment showing functional differences between groups. *P<0.05; **P<0.01; ***P<0.001. PCD, Programmed Cell Death; MCPcounter, Microenvironment Cell Populations counter; CIBERSORT, Cell-type Identification By Estimating Relative Subsets Of RNA Transcripts; QUANTISEQ, Quantification of the Tumor Immune Contexture from Human RNA-seq data; EPIC, Estimating the Proportions of Immune and Cancer cells; TIMER, Tumor Immune Estimation Resource; ESTIMATE, Estimation of Stromal and Immune cells in Malignant Tumor tissues using Expression data; GSEA, Gene Set Enrichment Analysis.

To characterize the immune landscape associated with the cell death–based risk signature, we applied six deconvolution algorithms to assess immune cell infiltration in melanoma (**Figures 6A–F**). Despite methodological heterogeneity, the majority of algorithms converged on a consistent pattern: the low-risk group exhibited heightened infiltration of antitumor effector populations, including CD8^+^ T cells, M1-polarized macrophages, and NK cells. In contrast, the high-risk group was characterized by elevated infiltration of M2 macrophages, reflecting a protumor genic and immunosuppressive phenotype. Additionally, the EPIC algorithm uniquely revealed a significant enrichment of cancer-associated fibroblasts (CAFs) in high-risk tumors. Kaplan–Meier analysis confirmed that elevated levels of M0/M2 macrophages and resting NK cells were significantly associated with poorer OS, whereas higher infiltration of M1 macrophages, CD8^+^ T cells, and CD4^+^ memory-activated T cells correlated with favorable prognosis (p < 0.05, **Figure 6G**). Moreover, immune microenvironment profiling via the ESTIMATE algorithm further substantiated these observations: high-risk tumors exhibited significantly higher tumor purity, coupled with markedly decreased stromal and immune scores, reflecting a more immune-depleted and stroma-deficient microenvironment (**Figure 6H**). We further examined the expression landscape of immune checkpoint and immunomodulatory genes. The low-risk group exhibited broadly elevated expression of immune-related genes, including those involved in T cell activation and antigen presentation (**Figure 6I**). GSEA enrichment analysis corroborated these findings, with immune-associated pathways (e.g., interferon response, T cell receptor signaling, and MHC-mediated antigen processing) predominantly enriched in the low-risk group. In contrast, the high-risk group showed significant enrichment of oncogenic and metabolic programs, including glycolytic metabolism, Wnt/β-catenin signaling, Notch pathway activation, and DNA repair machinery (**Figure 6J**).

### 3.7 Genomic alterations, and ICI response prediction inferred from a cell death–linked risk signature

**Figure 7:**
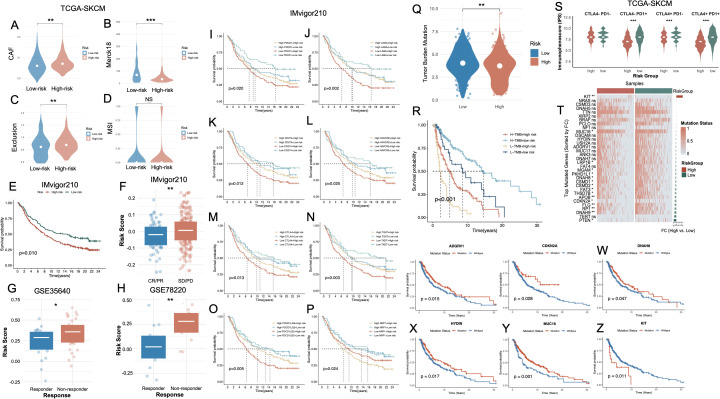
Association of the PCD-based risk signature with immune characteristics, immunotherapy response, and mutation status in melanoma. **(A–D)** Violin plots CAF, Merck18 immune-related score, T cell exclusion, and MSI between high- and low-risk groups in the TCGA-SKCM cohort. **(E)** Kaplan–Meier survival curve showing significantly worse survival in the high-risk group of the IMvigor210 cohort. **(F)** Boxplot comparing risk scores between high- and low-risk groups in IMvigor210, confirming elevated scores in high-risk patients. **(G, H)** Risk scores are significantly higher in non-responders vs. responders to immunotherapy in the GSE35640 and GSE78220 cohorts. **(I–P)** Kaplan–Meier survival analyses in the IMvigor210 cohort, stratified by immune checkpoint gene expression levels and risk group. **(Q)** Violin plot showing TMB is significantly higher in high-risk patients. **(R)** Kaplan–Meier curve indicating that combined high TMB and low risk score predicts the best survival outcomes, whereas low TMB and high risk confers the poorest prognosis. **(S)** Violin plots of IPS across CTLA4/PD1 combinations. **(T)** Heatmap of top mutated genes sorted by fold change between high- and low-risk groups in TCGA-SKCM. **(U–Z)** Kaplan–Meier survival curves comparing mutation vs. wild-type status of representative genes. *P<0.05; **P<0.01; ***P<0.001. PCD, Programmed Cell Death; CAF, Cancer-Associated Fibroblast; MSI, Microsatellite Instability; TMB, Tumor Mutational Burden; IPS, Immunophenoscore; CR, Complete Response; PR, Partial Response; SD, Stable Disease; PD, Progressive Disease; TCGA, The Cancer Genome Atlas; SKCM, Skin Cutaneous Melanoma.

To further investigate the immunological implications of the cell death-based risk model, we analyzed distinct tumor microenvironment features in the TCGA-SKCM cohort. High-risk tumors exhibited significantly elevated CAF (**Figure 7A**, p < 0.01). Additionally, Merck18 expression was markedly higher in the high-risk group (**Figure 7B**, p < 0.001). The Exclusion score was also significantly elevated in high-risk tumors (**Figure 7C**, p < 0.01). In contrast, no significant differences in MSI were observed between groups (**Figure 7D**, NS). Validation in three independent ICB-treated melanoma cohorts—IMvigor210, GSE35640, and GSE78220—further substantiated the prognostic and predictive utility of the cell death–associated risk model. In the IMvigor210 cohort, high-risk patients exhibited significantly inferior OS compared to their low-risk counterparts (p = 0.010, **Figure 7E**), alongside markedly elevated risk scores (**Figure 7F**). Similarly, in the GSE35640 dataset, non-responders to ICB therapy displayed substantially higher risk scores relative to responders (**Figure 7G**). The GSE78220 cohort yielded consistent results, with high-risk individuals showing not only significantly higher risk scores (p < 0.01, **Figure 7H**) but also strong enrichment in the non-responder group. Prognostic evaluation stratified by the expression of immune checkpoint–related genes revealed that elevated risk scores uniformly predicted unfavorable survival, irrespective of PDCD1, LAG3, CD274, CTLA4, HAVCR2, CTLA4, TIGIT, PDCD1LG2 or NRP1 expression levels (**Figures 7I-P**, respectively). TMB analysis revealed significantly higher TMB levels in the low-risk group (**Figure 7Q**), and survival analyses demonstrated that patients with high TMB and low risk had the most favorable prognosis, while high TMB did not rescue poor outcomes in the high-risk group (**Figure 7R**, **Supplementary Figure S4A**).

We subsequently interrogated the predictive utility of the cell death–associated risk model in the context of immune checkpoint inhibitor (ICI) therapy. Immunophenoscore (IPS) stratification under all four combinatorial checkpoint statuses (CTLA4^+^/^−^ and PD1^+^/^−^) consistently revealed significantly elevated immunogenic potential in the low-risk group, independent of checkpoint molecule expression levels (**Figure 7S**). The overall mutational landscape of the SKCM cohort was presented in **Supplementary Figure S4B**. Comprehensive somatic mutational profiling further demonstrated a markedly elevated TMB in the low-risk cohort. Several frequently mutated genes—such as CDKN2A, RP1, FLG, APOB, PTEN, and FAT3—exhibited significantly higher mutation frequencies in low-risk tumors (**Figure 7T**). Notably, these alterations were predominantly enriched in the low-risk population, potentially reflecting a more expansive neoantigenic repertoire that may potentiate enhanced tumor immunogenicity and heightened responsiveness to immunotherapy. Survival analyses further revealed that, with the exception of KIT, mutations in ADGRV1, CDKN2A, DNAH8, HYDIN, and MUC16 (**Figures 7U-Y**, respectively) were significantly associated with favorable OS. Conversely, KIT mutations were linked to dismal prognoses (**Figure 7Z**).

### 3.8 Cell-type specific transcriptomic modeling reveals robust diagnostic potential of a cell death–associated gene signature

**Figure 8:**
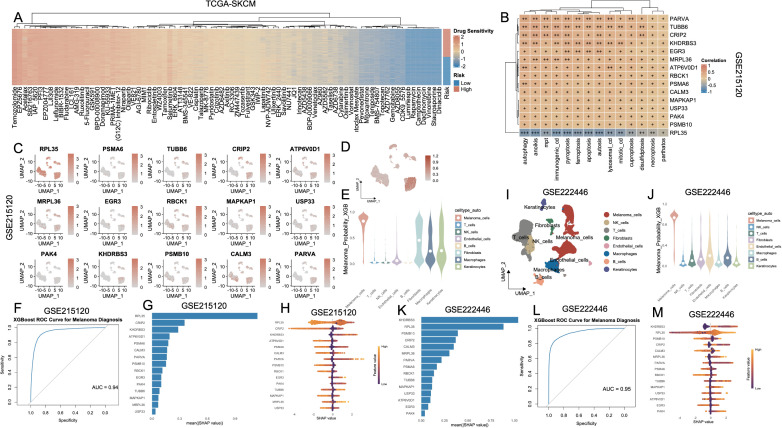
Identification and validation of melanoma cell–associated diagnostic markers based on PCD-related features and machine learning. **(A)** Heatmap showing the drug sensitivity landscape of melanoma patients (TCGA-SKCM) stratified by PCD-based risk groups. Rows represent drugs, and columns are patients ordered by risk. **(B)** Correlation heatmap of key diagnostic genes and PCD pathway scores in the TCGA-SKCM cohort. **(C)** UMAP plots showing the expression patterns of 15 signature genes in the GSE215120 cohort. **(D)** XGBoost-predicted melanoma probability score projected onto the UMAP of GSE215120, indicating high specificity for melanoma cells. **(E)** Violin plot of XGBoost-predicted melanoma probability across different cell types, demonstrating that prediction is specific to melanoma cells in GSE215120. **(F)** ROC curve showing the high diagnostic accuracy of the XGBoost model in distinguishing melanoma cells in GSE215120. **(G, H)** Feature importance plots (mean (|SHAP value|) and SHAP summary) for top predictors in the GSE215120 cohort. **(I)** UMAP visualization of cell types in the external validation cohort GSE222446. **(J)** Violin plot of melanoma prediction probability in GSE222446. **(K–M)** Feature importance analysis and SHAP summary plots in GSE222446. **(L)** ROC curve of the XGBoost model in GSE222446, demonstrating high diagnostic power. PCD, Programmed Cell Death; TCGA, The Cancer Genome Atlas; SKCM, Skin Cutaneous Melanoma; UMAP, Uniform Manifold Approximation and Projection; XGBoost, eXtreme Gradient Boosting; SHAP, SHapley Additive exPlanations; ROC, Receiver Operating Characteristic.

A heatmap of significantly differential compounds (p < 0.0001) revealed distinct drug response profiles (**Figure 8A**). High-risk and low-risk groups exhibited distinct drug sensitivity profiles (**Supplementary Figure S5**, **Supplementary Table S6**). Clinical correlation analysis revealed that high-risk patients exhibited markedly elevated proportions of advanced disease characteristics, including T3-T4 lesions, nodal involvement (N+), distant metastases (M1), and stage III-IV classifications, relative to their low-risk counterparts (**Supplementary Figure S6**). Next, we analyzed the relationship between the 15 key genes and cell death modalities within the GSE215120 single-cell dataset. Spearman correlation analysis demonstrated strong positive associations between many genes (e.g., PARVA, TUBB6, KHDRBS3, MRPL36) and multiple cell death types, particularly autophagy, anoikis, immunogenic cell death and ferroptosis (**Figure 8B**). The association between model gene expression and the activity of PCD in AM3_post and CM1_lym was shown in **Supplementary Figures S7A, B**. To further explore the biological relevance of the 15-gene panel, we performed GO enrichment analysis. The results showed that these genes were significantly enriched in cellular component–related terms such as proteasome core complex, sarcomere, myofibril, ribosome, contractile muscle fiber, proteasome complex, and endopeptidase complex (**Supplementary Table S7**). UMAP expression plots confirmed that these genes were predominantly expressed in melanoma cells compared to non-malignant compartments (**Figure 8C**). A composite UMAP feature plot summarizing the integrated expression landscape across all 15 genes reaffirmed their restriction to malignant clusters (**Figure 8D**). Based on these features, we trained an XGBoost classifier to distinguish melanoma cells from non-malignant cells using single-cell transcriptomes. The violin plot of melanoma probability scores revealed a clear separation between melanoma and stromal/immune cell populations (**Figure 8E**). Model performance was evaluated using a ROC curve, which yielded an AUC of 0.94 (**Figure 8F**). SHAP value analysis ranked RPL35, KHDRBS3, ATP6V0D1, and CRIP2 as the most important contributors to melanoma classification (**Figure 8G**). The SHAP summary plot (**Figure 8H**) further revealed that higher expression of these genes consistently drove classification toward the melanoma phenotype. To externally validate these findings, we applied the same model to an independent single-cell melanoma dataset (GSE222446). UMAP clustering (**Figure 8I**) and melanoma probability scores (**Figure 8J**) recapitulated the separation between malignant and non-malignant compartments. The classifier maintained robust predictive performance with an AUC of 0.95 (**Figure 8L**). SHAP importance rankings in this cohort also confirmed KHDRBS3, RPL35, CALM3, and PSMB10 as top diagnostic features (**Figures 8K–M**).

## 4 Discussion

In this study, we present a comprehensive single-cell and bulk transcriptomic investigation of PCD landscapes in melanoma, uncovering their functional relevance in tumor progression, immune evasion, and response to ICB. By integrating AUCell-based scoring, unsupervised clustering, and intercellular communication profiling, we delineated the heterogeneity of PCD activation across malignant and stromal compartments, providing a refined molecular stratification of melanoma at the cell-state level.

Among the diverse PCD modalities profiled, disulfidptosis, ICD, and autosis emerged as the most prominently activated programs within melanoma cells. Disulfidptosis is a novel form of programmed cell death triggered by glucose starvation, in which SLC7A11 overexpression depletes NADPH and accumulates cystine, leading to aberrant disulfide bonding, actin cytoskeleton disruption, and cell collapse (28). Previous studies have demonstrated that disulfidptosis is intricately associated with adverse oncologic outcomes, encompassing poor cancer prognosis, an immunosuppressive tumor microenvironment (TME), heightened immune evasion, and diminished therapeutic responsiveness across multiple malignancies, including renal, colorectal, bladder carcinomas, and melanoma (29, 30). Although ICD has been proposed to enhance antitumor immunity and improve prognosis in melanoma (31), our study paradoxically found elevated ICD activity in melanoma cells to be associated with advanced clinical stage. We speculate that this may reflect a non-productive ICD phenotype, wherein tumor cells emit ICD-associated signals (e.g., calreticulin, HMGB1) but fail to initiate effective immune clearance due to a profoundly immunosuppressive microenvironment. Prior studies have demonstrated that chronic inflammation, T cell dysfunction, and impaired dendritic cell maturation in late-stage tumors can hinder the translation of ICD signals into productive antitumor immunity (32, 33). In parallel, autosis—a Na^+^/K^+^-ATPase–regulated, autophagy-dependent cell death modality—was markedly elevated in melanoma cells, particularly in later-stage disease and younger patients. Autosis is known to be activated by metabolic stressors such as hypoxia, nutrient deprivation, and excessive autophagic flux—conditions frequently observed in highly proliferative and metabolically demanding melanoma subsets. These findings suggest that autosis may operate as a stress-adaptive death mechanism that accompanies, or possibly contributes to, melanoma progression and cellular aggressiveness (34).

Recent evidence has elucidated a pivotal role of ferroptosis in modulating therapeutic resistance in BRAF-mutant melanoma (35). Although BRAF inhibitors such as vemurafenib can initially induce ferroptosis-like cell death through lipid peroxidation, tumor cells often adapt by upregulating antioxidant defenses, particularly the SLC7A11–GPX4 axis. This adaptation suppresses ferroptotic cell death and contributes to acquired resistance. Notably, pharmacologic inhibition of GPX4 or glutathione synthesis has been shown to restore ferroptosis sensitivity and resensitize melanoma cells to BRAF-targeted therapy (36). In addition, BRAF inhibition has been found to induce adaptive autophagy in BRAF-mutant melanoma, which facilitates tumor cell survival under therapeutic pressure (37). Co-inhibition of autophagy synergizes with BRAF inhibition to enhance cell death, highlighting autophagy as a therapeutically actionable resistance mechanism in this context. Beyond ferroptosis and autophagy, necroptosis has also emerged as a potential vulnerability. In BRAF(V600E)-mutant melanoma cells, dabrafenib was shown to suppress necroptotic signaling pathways (38), suggesting that activating necroptosis may circumvent resistance mechanisms. Moreover, pyroptosis has been implicated as a promising adjunct strategy. In BRAF-mutant melanoma, pharmacologic induction of gasdermin D/E-mediated pyroptosis has demonstrated synergy with BRAF/MEK inhibition in resistant models, eliciting immunogenic cell death and potentiating anti-tumor immunity (39). These findings underscore the therapeutic potential of leveraging PCD pathways to enhance the efficacy of BRAF-targeted treatments and overcome resistance in BRAF-mutant melanoma.

Notably, among all cell types profiled, melanoma cells exhibited the highest median activity across multiple PCD modalities, surpassing that of immune effectors such as T cells and NK cells, as well as stromal constituents including fibroblasts and endothelial cells. This heightened death pathway activation underscores the unique stress-adaptive phenotype of melanoma cells, which appear to maintain viability despite the constitutive engagement of pro-death programs. This paradox may reflect a state of homeostatic tension, wherein melanoma cells operate near the threshold of lethal stress, counterbalanced by robust survival mechanisms—such as hyperactivated antioxidant pathways, metabolic rewiring, and autophagy buffering. The relatively lower PCD activity observed in T cells and fibroblasts, by contrast, suggests a more quiescent or regulated death profile, potentially optimized for immune effector function or stromal maintenance, respectively. In melanoma, the simultaneous activation of multiple PCD pathways may be driven by chronic exposure to oxidative stress, hypoxia, nutrient limitation, or immune-mediated pressure—all hallmarks of the tumor microenvironment. However, the failure of these death signals to culminate in effective tumor cell clearance highlights the ability of melanoma cells to co-opt cell death pathways, not as terminal endpoints, but as adaptive stress circuits (40). Following anti-PD1 therapy, we observed a marked attenuation in PCD activity across the vast majority of cellular compartments. This systemic reduction may indicate that immune checkpoint blockade indirectly perturbs tumor-intrinsic death programs, potentially via the alleviation of cytotoxic lymphocyte-driven selective pressures or the establishment of a post-therapeutic immunologically exhausted state (41). Intriguingly, lymphatic metastatic lesions exhibited globally elevated PCD activities compared to their primary tumor counterparts. Rather than simply indicating increased immune-mediated clearance, this elevation may signify adaptive responses to the unique immunometabolic pressures of the lymphatic niche—such as hypoxia, altered nutrient availability, and enriched stromal interactions—which may potentiate compensatory death pathway activation. From a clinical perspective, we observed that patients with stage IV melanoma exhibited significantly higher PCD activity compared to those in stage III, suggesting a potential role for death pathway engagement in aggressive or treatment-refractory disease states. Moreover, patients under 60 years of age demonstrated consistently higher levels of PCD across modalities. This may imply that younger individuals possess greater immune vigor or transcriptional plasticity, enabling them to sustain heightened stress-adaptive and apoptotic responses. Accordingly, the elevated cell death activity observed in younger patients may not merely indicate increased vulnerability, but rather reflect a more proliferative, metabolically active, or immunologically sculpted tumor context shaped by dynamic host–tumor interactions.

Subtype-level dissection of fibroblasts and immune cells revealed further complexity. Among fibroblast subsets, apCAFs demonstrated the highest overall activation of PCD pathways. This finding is biologically plausible given the immunologically active phenotype of apCAFs, which are characterized by expression of MHC class II molecules and the ability to interact with CD4^+^ T cells via antigen presentation (42). Notably, apCAFs are thought to arise from mesenchymal precursors that acquire immune-like features through IFN-γ signaling (43). Such phenotypic plasticity, while potentially facilitating immune modulation, may also sensitize these cells to stress-induced cell death processes such as ferroptosis or immunogenic cell death. In parallel, vCAFs harbored markedly elevated PCD activity, which may correspond to their high metabolic turnover and involvement in extracellular matrix remodeling and angiogenesis. These functional characteristics, including strong associations with epithelial-mesenchymal transition (EMT), cell adhesion regulation, collagen fibril organization, and key signaling cascades such as Wnt and TGF-β, underscore the pivotal role of vCAFs in orchestrating tumor vascularization, stromal reprogramming, cellular migration, and the acquisition of invasive phenotypes (44). The heightened activation of PCD pathways in vCAFs may reflect a compensatory response to their sustained bioenergetic demands and dynamic interactions within the tumor microenvironment. Within the T and NK cell compartments, CD8_CM and proliferative NK_MKI67 subsets exhibited markedly elevated levels of PCD activity compared to their CD4_EM and NK_FCGR3A counterparts, respectively. Notably, CD4_REG/Treg also demonstrated a relatively high degree of PCD activation. This observation is biologically consistent with prior studies indicating that, despite their immunosuppressive function, Tregs are not inherently resistant to cell death (45). In fact, tumor-infiltrating Tregs are frequently subjected to chronic TCR stimulation and reduced IL-2 availability—conditions that favor the upregulation of pro-apoptotic molecules such as BIM, rendering these cells susceptible to apoptosis (46). These observations suggest that cytotoxic and proliferative lymphocyte subsets may be more susceptible to activation-induced cell death (AICD), potentially reflecting an immunoregulatory mechanism designed to constrain excessive effector responses. However, despite this elevated PCD activity, downstream analyses revealed a significant inverse correlation between the activation of most cell death pathways and the expression of T and NK cell functional signatures—including TCR signaling, cytotoxic effector functions, and cytokine-mediated immune activation. This paradox likely reflects a progressive trajectory of functional exhaustion and terminal differentiation, wherein sustained antigenic stimulation and metabolic stress induce high PCD engagement concomitant with the erosion of immune competency. Persistent antigen exposure and metabolic stress have been implicated in driving T cell exhaustion and apoptosis, undermining antitumor immunity (47). Likewise, chronic activation renders NK cells functionally impaired and prone to cell death within the tumor microenvironment (48), supporting an activation-exhaustion axis underlying elevated PCD in cytotoxic lymphocytes.

Strikingly, when stratifying melanoma cells by global PCD activity (across all 13 modalities), we observed that cells in the high-activity group (Melanoma_High) vastly outnumbered those in the low-activity group. This disproportionate distribution suggests that PCD hyperactivation may be a dominant phenotype in melanoma evolution, potentially driven by selective pressures that favor apoptotic escape and immune resistance. Transcriptomic analysis of Melanoma_High cells revealed upregulation of invasive and epigenetic regulators (e.g., AXL, EZH2, KDM5A), as well as enriched pathways involved in NF-κB, TNF, and IL6-JAK-STAT3 signaling. These features, together with higher inferred CNV burden and elevated interaction with fibroblasts and endothelial cells, support the notion that Melanoma_High cells represent a transcriptionally reprogrammed, genomically unstable, and microenvironmentally engaged malignant subpopulation with aggressive clinical behavior. Consistent with this, the PCD–associated risk signature constructed from 15 prognostic genes robustly stratified patients into high- and low-risk groups with distinct immune microenvironment features. The high-risk group exhibited suppressed antitumor immunity, characterized by elevated M2 macrophage infiltration, reduced CD8^+^ and M1 macrophages, and lower stromal and immune scores. Functionally, GSEA revealed that high-risk tumors were enriched for oncogenic pathways including glycolysis, Wnt/β-catenin, Notch, and DNA repair, whereas low-risk tumors showed upregulation of immune-activating pathways such as interferon signaling and antigen presentation. Importantly, risk score also proved predictive of ICI responsiveness. In multiple ICB-treated cohorts, non-responders consistently exhibited higher risk scores. Moreover, low-risk tumors were associated with significantly higher TMB, elevated IPS, and favorable response across various checkpoint blockade combinations. Somatic mutations enriched in the low-risk group (e.g., CDKN2A, MUC16, DNAH8) may reflect increased neoantigen load, enhancing tumor visibility to the immune system.

To better understand the biological implications of the 15-gene model, we conducted GO enrichment analysis and found strong enrichment in pathways related to proteasomal degradation, ribosome structure, and cytoskeletal organization. Notably, several top-ranked GO terms such as “proteasome core complex,” “ribosome,” and “contractile muscle fiber” suggest a potential involvement of the 15-gene panel in essential cellular infrastructure and regulatory mechanisms. Among these, the enrichment in proteasome-related components points to a functional connection between proteasome activity and cell death regulation. Beyond protein turnover, proteasome-mediated degradation has been implicated in modulating apoptotic pathways (49). Depending on the cellular context, proteasome inhibition can either promote apoptosis—via accumulation of pro-apoptotic regulators like p27Kip1—or inhibit it by stabilizing anti-apoptotic factors such as caspase inhibitors.

Lastly, the 15-gene signature demonstrated remarkable diagnostic utility. The enrichment of advanced AJCC stage characteristics among high-risk individuals underscores the model’s potential utility in reflecting disease aggressiveness. Furthermore, these genes showed strong correlations with multiple PCD modalities and exhibited melanoma-specific expression patterns in single-cell transcriptomic analyses. An XGBoost classifier trained on these genes achieved AUCs > 0.94 in two independent datasets, confirming their discriminative power for malignant cell identification. SHAP analysis identified RPL35, KHDRBS3, and ATP6V0D1 as top contributors, suggesting potential utility as biomarkers or therapeutic targets.

In conclusion, our findings reveal that cell death pathways are deeply interwoven with melanoma progression, immunoevasion, and therapeutic response. Beyond prognostic stratification, PCD profiling may serve as a foundation for identifying vulnerable cell states and guiding rational immunotherapeutic strategies. Future work integrating single-cell transcriptomics and lineage tracing will be instrumental in defining the causal relationships between cell death dynamics, immune contexture, and tumor evolution.

Despite the comprehensive and integrative framework of this study, several limitations should be acknowledged. First, the inference of PCD activity was based on AUCell scoring of predefined gene sets, which may not fully capture the complexity of post-transcriptional and post-translational regulation. Second, the immunotherapy-related predictions were mainly inferred from computational models—including IPS, TMB, and risk stratification—and thus warrant validation in prospective clinical cohorts. Moreover, the current model does not differentiate between distinct classes of immune checkpoint inhibitors (e.g., anti–PD-1 vs. anti–CTLA-4). Third, this study is grounded in computational analyses of single-cell transcriptomic data and does not currently include direct experimental validation. Although our findings highlight distinct PCD signatures across stromal and immune subsets in melanoma, the mechanistic roles of specific PCD-related pathways—such as disulfidptosis—remain to be functionally interrogated. Future studies incorporating gene perturbation strategies (e.g., CRISPR-mediated knockout or ectopic expression) and protein-level assays (such as immunohistochemistry or immunoblotting) will be instrumental in substantiating the biological significance of our observations. Furthermore, as the prognostic model was developed and validated using public datasets, its broader clinical applicability should ideally be confirmed in future prospective, multicenter studies.

## 5 Conclusion

Our study provides a comprehensive single-cell framework for understanding the landscape and clinical implications of PCD in melanoma. We demonstrate that elevated cell death activity is intricately linked to tumor progression, immune modulation, and therapeutic response. These findings highlight the prognostic and diagnostic utility of PCD-related transcriptional programs and offer new insights into the cellular dynamics underpinning melanoma aggressiveness.

## Data Availability

The datasets presented in this study can be found in online repositories. The names of the repository/repositories and accession number(s) can be found in the article/**Supplementary Material**.

## Supplementary material

Supplementary Figure 1 Schematic overview of the study design integrating single-cell and bulk transcriptomic data to construct and validate a melanoma-specific PCD-related gene signature.

Supplementary Figure 2 Quality control, dimensionality reduction, and highly variable gene identification in melanoma single-cell datasets. **(A)** Violin plots showing distributions of RNA feature counts (nFeature_RNA), total transcript counts (nCount_RNA), and mitochondrial gene percentages (percent.mt) across annotated clusters in the integrated melanoma single-cell RNA-seq dataset (AM1–AM2, AM3_pre, AM4-AM6, CM1–CM3). **(B)** Scatter plots of average gene expression vs. variance to identify highly variable genes, highlighting top genes with the greatest standardized variance. **(C)** Correlation between transcript counts and gene features across clusters, with strong positive correlation coefficients labeled. **(D)** Elbow plot used to determine the optimal number of PCs to retain for downstream dimensionality reduction. **(E)** PCA projection of all cells, color-coded by identity, displaying overall transcriptomic heterogeneity across melanoma clusters. **(F)** PCA loading plots identifying top genes contributing to the first four principal components. **(G–I)** Comparison between AM3_post and CM1_lym subgroups, including PCA projection **(G)**, QC metrics **(I)**, and correlation plots. **(H)** Highly variable gene selection results specific to the AM3_post vs. CM1_lym dataset. **(J)** Violin plots showing RNA quality metrics for the AM3_post and CM1_lym subsets. **(K)** PCA loadings of top contributing genes for PC1–PC4 in the subsetted comparison between AM3_post and CM1_lym. **(L)** Elbow plot for PC selection in the subsetted data. PC, Principal Component; PCA, Principal Component Analysis.

Supplementary Figure 3 Single-cell distribution of 13 programmed cell death types across tumor, stromal and immune compartments in the melanoma microenvironment.

Supplementary Figure 4 Survival significance and mutational landscape associated with TMB in melanoma. **(A)** Kaplan–Meier survival analysis comparing overall survival between high TMB (H–TMB) and low TMB (L–TMB) groups in the TCGA-SKCM cohort. **(B)** Waterfall plot showing the top 40 most frequently mutated genes across 469 melanoma samples in TCGA-SKCM. Mutation types include missense, nonsense, frameshift, and splice site variants. The upper bar graph shows total TMB per sample, and the right bar graph shows the number and percentage of samples mutated per gene. TMB, Tumor Mutational Burden; H–TMB, High Tumor Mutational Burden; L–TMB, Low Tumor Mutational Burden; TCGA, The Cancer Genome Atlas; SKCM, Skin Cutaneous Melanoma.

Supplementary Figure 5 Comparison of drug sensitivity between high- and low-risk groups based on the PCD-related gene signature. Boxplots showing estimated IC50 values for various anticancer drugs across high-risk (red) and low-risk (blue) groups. PCD, Programmed Cell Death; IC50, Half-maximal Inhibitory Concentration.

Supplementary Figure 6 Distribution of clinical stage features between risk groups in the TCGA-SKCM cohort, stratified by the 15-gene prognostic model. Stacked bar plots show the proportional distribution of (from left to right): **(A)** AJCC_T stage (T1-T2 vs. T3-T4), **(B)** AJCC_N stage (N0 vs. N+), **(C)** AJCC_M stage (M0 vs. M1), and **(D)** overall clinical stage (Stage I+II vs. Stage III+IV) between low- and high-risk groups as defined by the 15-gene prognostic model. TCGA-SKAM, The Cancer Genome Atlas-Skin Cutaneous Melanoma; AJCC, American Joint Committee on Cancer.

Supplementary Figure 7 Correlation between diagnostic gene expression and PCD pathways in different melanoma subtypes. **(A, B)** Heatmaps showing Spearman correlation coefficients between the expression of 15 diagnostic genes and 13 PCD-related pathways across two melanoma subgroups: **(A)** CM_lym and **(B)** AM3_post. Positive correlations are shown in brown and negative correlations in blue, and “+” symbols denote correlation strength based on coefficient (ρ) thresholds: “+”: weak correlation (0 < ρ ≤ 0.3); “++”: moderate correlation (0.31 ≤ ρ ≤ 0.6); “+++”: strong correlation (ρ > 0.6). PCD, Programmed Cell Death; CM_lym, Cutaneous Melanoma (lymphoid subtype); AM3_post, Acral Melanoma (post-treatment subgroup).
