# Supplementary figures and images for: Programmed cell death-driven remodeling of the melanoma microenvironment enables prognostic stratification and therapeutic prediction
Source: Front Immunol. 2025 Aug 20;16:1612217. doi: 10.3389/fimmu.2025.1612217 (PMC12406059; doi:10.3389/fimmu.2025.1612217)

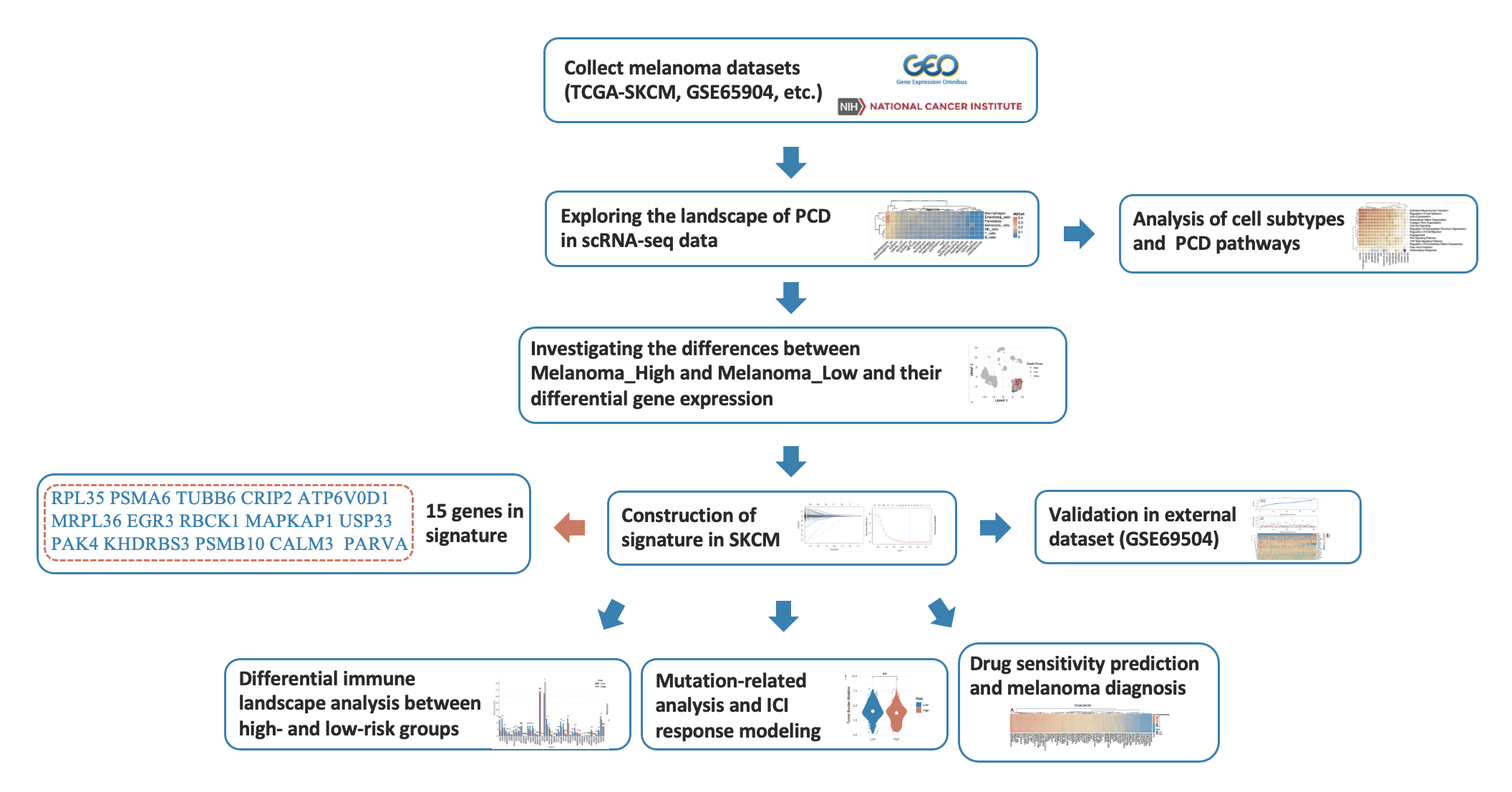

Supplement: Supplementary Figure 1 — Schematic overview of the study design integrating single-cell and bulk transcriptomic data to construct and validate a melanoma-specific PCD-related gene signature. [file Image1.png]

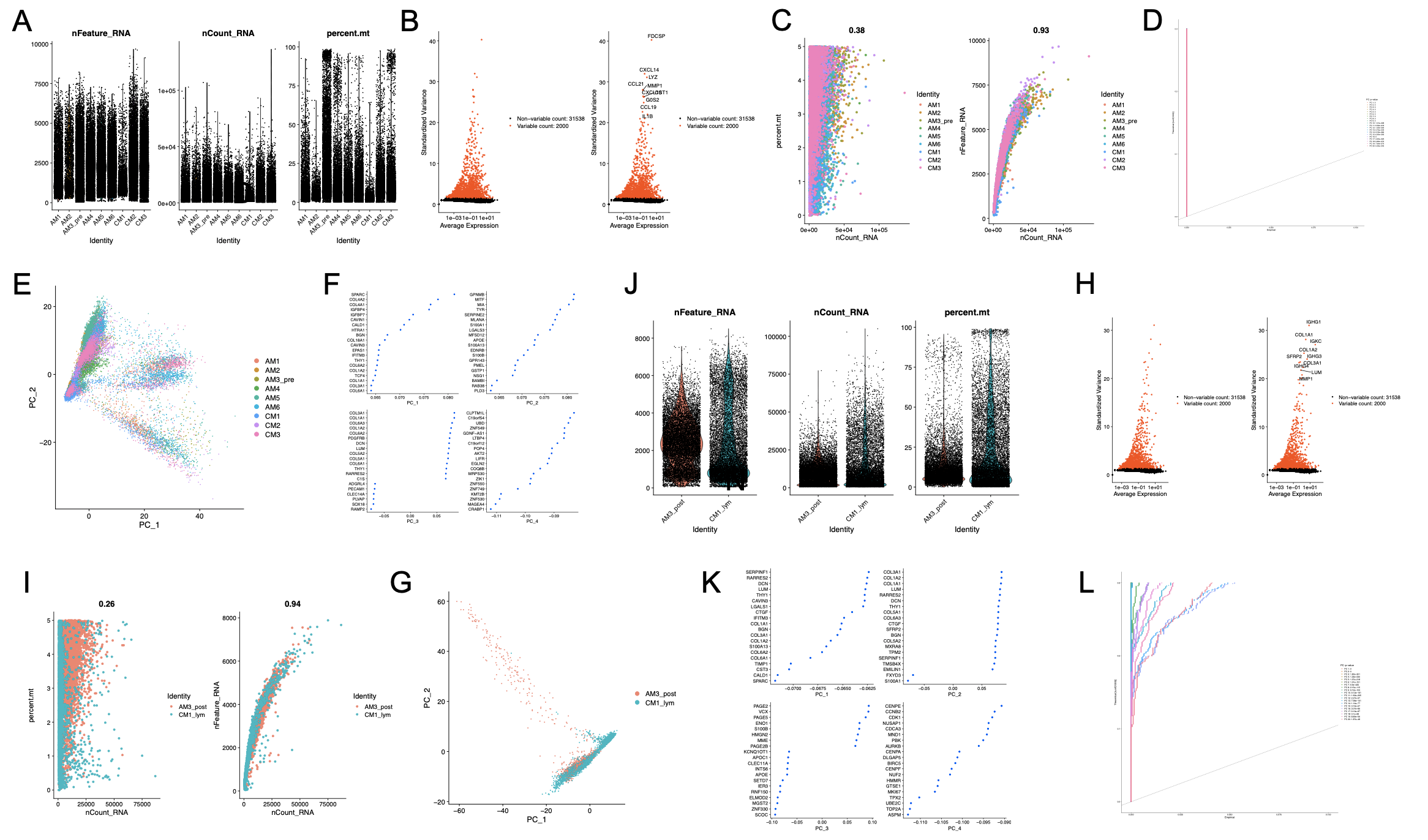

Supplement: Supplementary Figure 2 — Quality control, dimensionality reduction, and highly variable gene identification in melanoma single-cell datasets. (A) Violin plots showing distributions of RNA feature counts (nFeature_RNA), total transcript counts (nCount_RNA), and mitochondrial gene percentages (percent.mt) across annotated clusters in the integrated melanoma single-cell RNA-seq dataset (AM1–AM2, AM3_pre, AM4-AM6, CM1–CM3). (B) Scatter plots of average gene expression vs. variance to identify highly variable genes, highlighting top genes with the greatest standardized variance. (C) Correlation between transcript counts and gene features across clusters, with strong positive correlation coefficients labeled. (D) Elbow plot used to determine the optimal number of PCs to retain for downstream dimensionality reduction. (E) PCA projection of all cells, color-coded by identity, displaying overall transcriptomic heterogeneity across melanoma clusters. (F) PCA loading plots identifying top genes contributing to the first four principal components. (G–I) Comparison between AM3_post and CM1_lym subgroups, including PCA projection (G), QC metrics (I), and correlation plots. (H) Highly variable gene selection results specific to the AM3_post vs. CM1_lym dataset. (J) Violin plots showing RNA quality metrics for the AM3_post and CM1_lym subsets. (K) PCA loadings of top contributing genes for PC1–PC4 in the subsetted comparison between AM3_post and CM1_lym. (L) Elbow plot for PC selection in the subsetted data. PC, Principal Component; PCA, Principal Component Analysis. [file Image2.png]

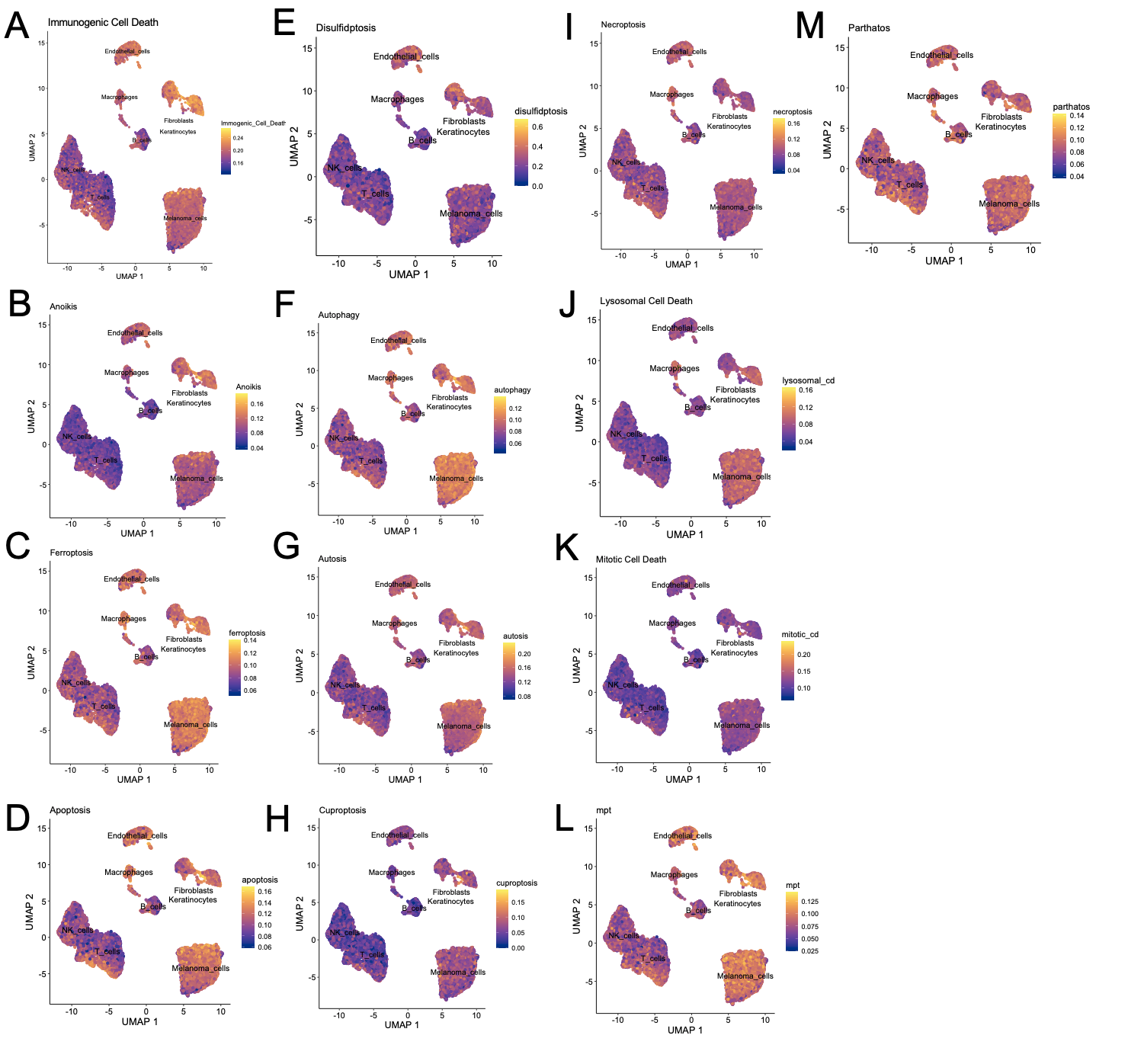

Supplement: Supplementary Figure 3 — Single-cell distribution of 13 programmed cell death types across tumor, stromal and immune compartments in the melanoma microenvironment. [file Image3.png]

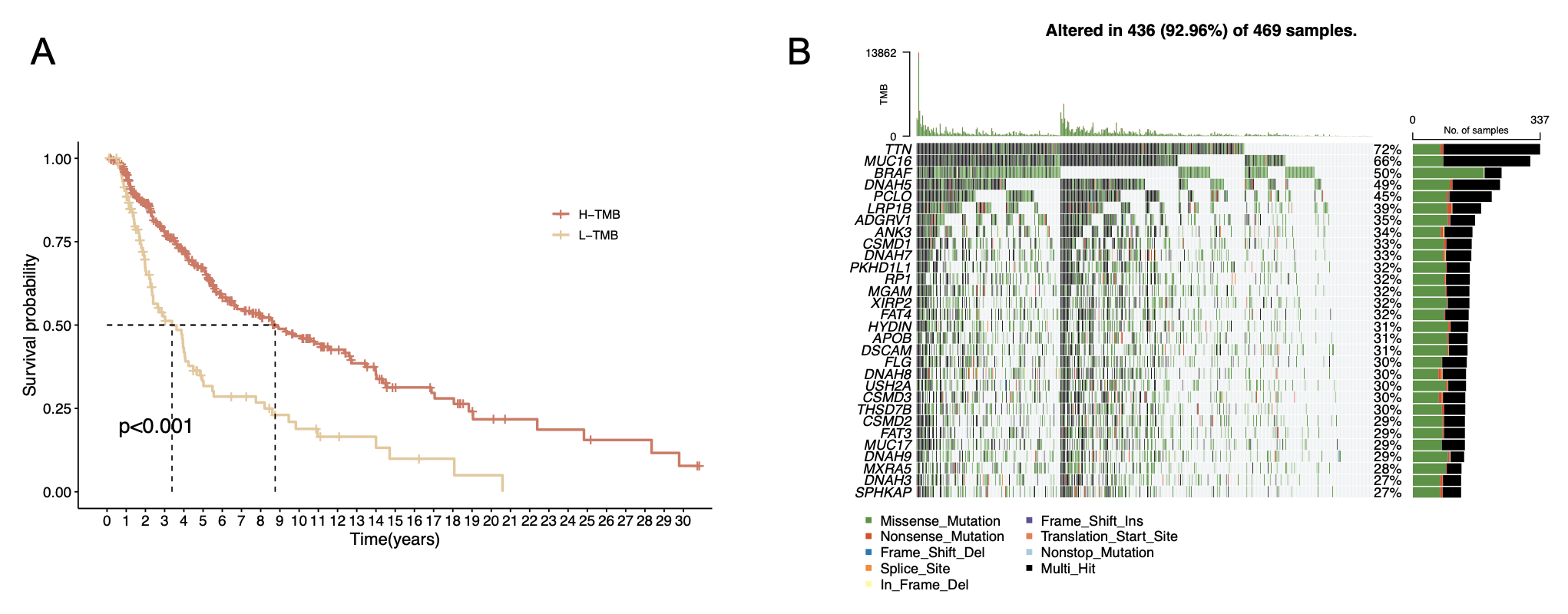

Supplement: Supplementary Figure 4 — Survival significance and mutational landscape associated with TMB in melanoma. (A) Kaplan–Meier survival analysis comparing overall survival between high TMB (H–TMB) and low TMB (L–TMB) groups in the TCGA-SKCM cohort. (B) Waterfall plot showing the top 40 most frequently mutated genes across 469 melanoma samples in TCGA-SKCM. Mutation types include missense, nonsense, frameshift, and splice site variants. The upper bar graph shows total TMB per sample, and the right bar graph shows the number and percentage of samples mutated per gene. TMB, Tumor Mutational Burden; H–TMB, High Tumor Mutational Burden; L–TMB, Low Tumor Mutational Burden; TCGA, The Cancer Genome Atlas; SKCM, Skin Cutaneous Melanoma. [file Image4.png]

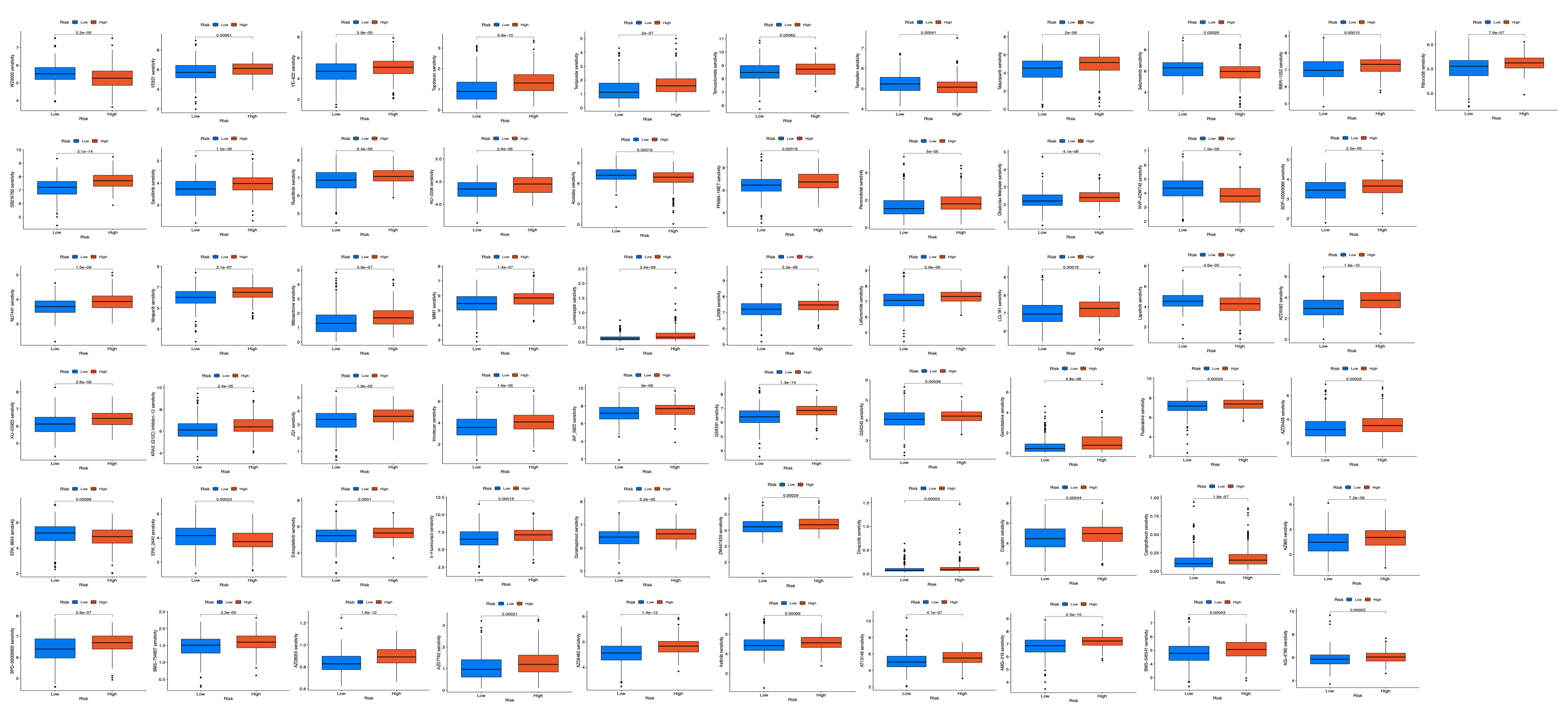

Supplement: Supplementary Figure 5 — Comparison of drug sensitivity between high- and low-risk groups based on the PCD-related gene signature. Boxplots showing estimated IC50 values for various anticancer drugs across high-risk (red) and low-risk (blue) groups. PCD, Programmed Cell Death; IC50, Half-maximal Inhibitory Concentration. [file Image5.png]

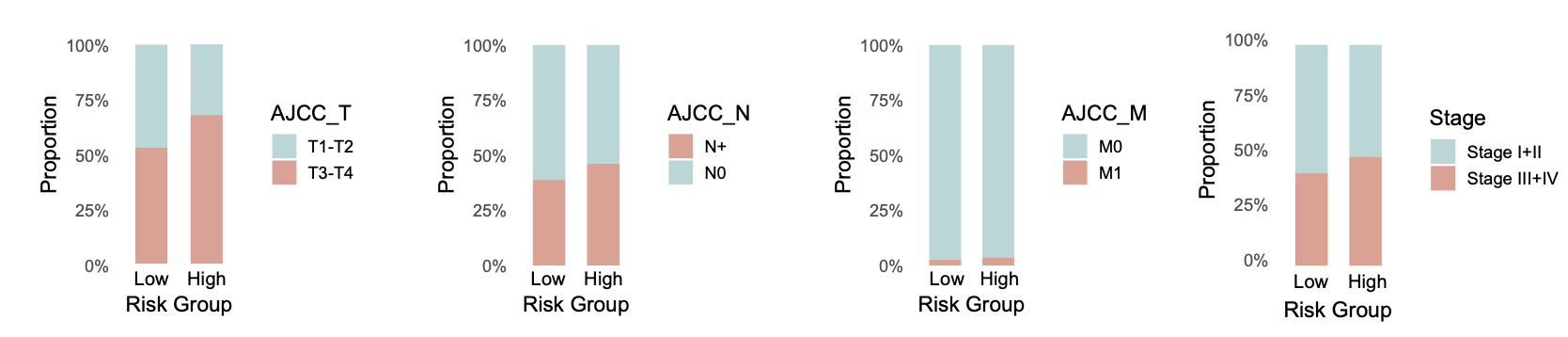

Supplement: Supplementary Figure 6 — Distribution of clinical stage features between risk groups in the TCGA-SKCM cohort, stratified by the 15-gene prognostic model. Stacked bar plots show the proportional distribution of (from left to right): (A) AJCC_T stage (T1-T2 vs. T3-T4), (B) AJCC_N stage (N0 vs. N+), (C) AJCC_M stage (M0 vs. M1), and (D) overall clinical stage (Stage I+II vs. Stage III+IV) between low- and high-risk groups as defined by the 15-gene prognostic model. TCGA-SKAM, The Cancer Genome Atlas-Skin Cutaneous Melanoma; AJCC, American Joint Committee on Cancer. [file Image6.png]

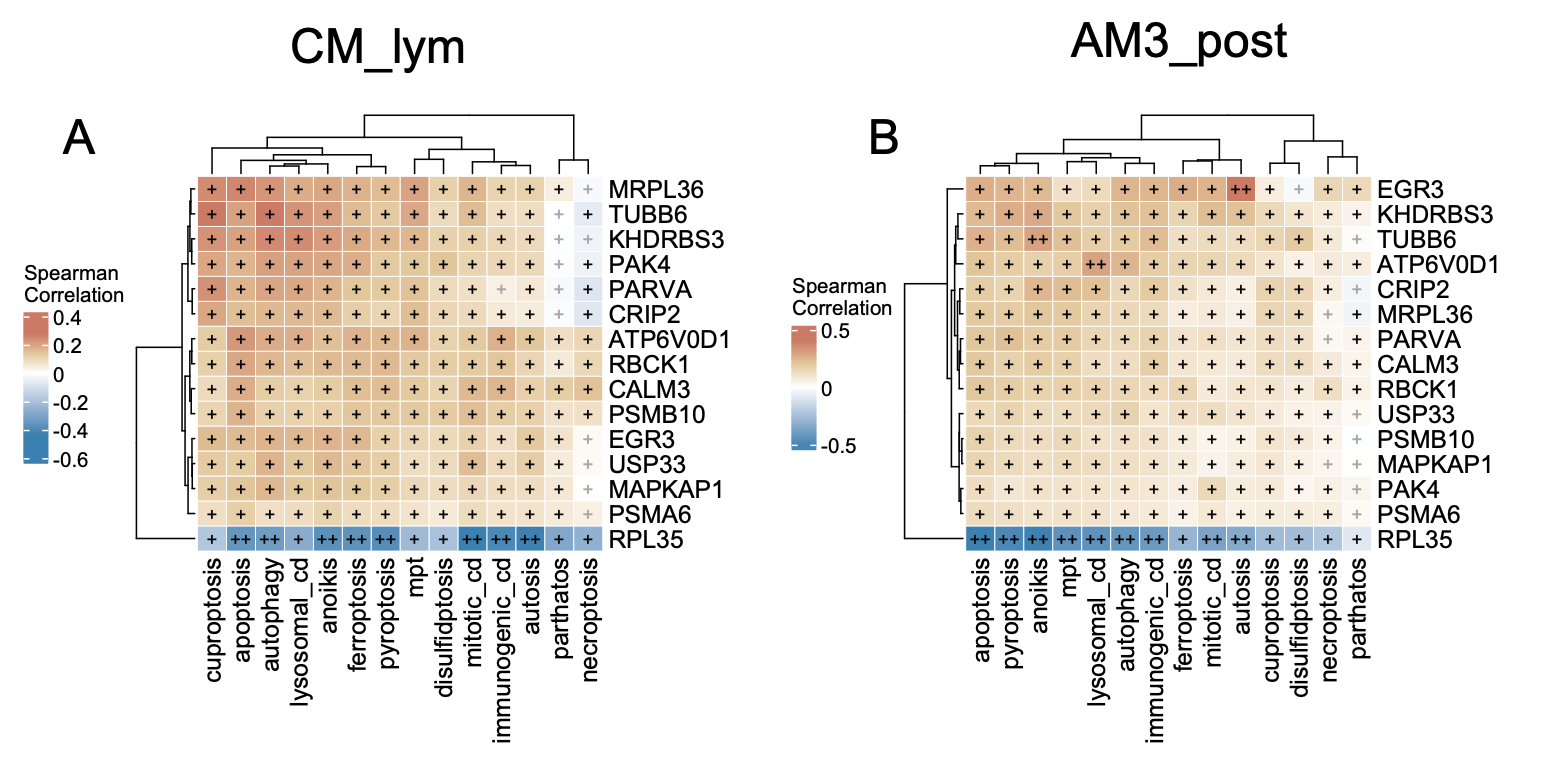

Supplement: Supplementary Figure 7 — Correlation between diagnostic gene expression and PCD pathways in different melanoma subtypes. (A, B) Heatmaps showing Spearman correlation coefficients between the expression of 15 diagnostic genes and 13 PCD-related pathways across two melanoma subgroups: (A) CM_lym and (B) AM3_post. Positive correlations are shown in brown and negative correlations in blue, and “+” symbols denote correlation strength based on coefficient (ρ) thresholds: “+”: weak correlation (0 < ρ ≤ 0.3); “++”: moderate correlation (0.31 ≤ ρ ≤ 0.6); “+++”: strong correlation (ρ > 0.6). PCD, Programmed Cell Death; CM_lym, Cutaneous Melanoma (lymphoid subtype); AM3_post, Acral Melanoma (post-treatment subgroup). [file Image7.png]
